# MAP3K1 regulates female reproductive tract development

**DOI:** 10.1242/dmm.050669

**Published:** 2024-03-28

**Authors:** Eiki Kimura, Maureen Mongan, Bo Xiao, Antonius Christianto, Jingjing Wang, Vinicius S. Carreira, Brad Bolon, Xiang Zhang, Katherine A. Burns, Jacek Biesiada, Mario Medvedovic, Alvaro Puga, Ying Xia

**Affiliations:** ^1^ Department of Environmental and Public Health Sciences, University of Cincinnati College of Medicine, Cincinnati, OH 45267-0056, USA; ^2^ GEMpath Inc., Longmont, CO 80501-1846, USA

**Keywords:** Female reproductive tract, Imperforate vagina, MAP3K1, Müllerian duct, WNT signaling

## Abstract

Mitogen-activated protein 3 kinase 1 (MAP3K1) has a plethora of cell type-specific functions not yet fully understood. Herein, we describe a role for MAP3K1 in female reproductive tract (FRT) development. MAP3K1 kinase domain-deficient female mice exhibited an imperforate vagina, labor failure and infertility. These defects corresponded with shunted Müllerian ducts (MDs), the embryonic precursors of FRT, that manifested as a contorted caudal vagina and abrogated vaginal–urogenital sinus fusion in neonates. The MAP3K1 kinase domain is required for optimal activation of the Jun-N-terminal kinase (JNK) and cell polarity in the MD epithelium, and for upregulation of WNT signaling in the mesenchyme surrounding the caudal MD. The MAP3K1-deficient epithelial cells and MD epithelium had reduced expression of WNT7B ligands. Correspondingly, conditioned media derived from MAP3K1-competent, but not -deficient, epithelial cells activated a TCF/Lef-luciferase reporter in fibroblasts. These observations indicate that MAP3K1 regulates MD caudal elongation and FRT development, in part through the induction of paracrine factors in the epithelium that trans-activate WNT signaling in the mesenchyme.

## Teaser

**Summary:** MAP3K1-deficient female mice display imperforate vagina and infertility due to the failure of Müllerian duct caudal elongation and its fusion with the urogenital sinus, and defective MAPK and WNT signaling.

## INTRODUCTION

The development of the mammalian reproductive system is a complex process with distinct sex-independent and -dependent phases. Prior to sexual differentiation, the reproductive tract precursors, known as the Müllerian (or paramesonephric) duct (MD) and the Wolffian (or mesonephric) duct (WD), develop in both male and female embryos. These ducts originate from the intermediate mesonephros and elongate caudally towards the urogenital sinus (UGS) (Orvis and Behringer, 2007). For male mice, the WD reaches and fuses with the UGS at embryonic day (E) 11.5, before developing into the internal genitalia, whereas for female mice, the MD fuses with the UGS later at E13.5 and ultimately forms the internal genitalia (Mullen and Behringer, 2014). The WD also has a role in the guidance of MD development.

Mouse sexual differentiation starts at E11.5, when the gonads in male embryos begin to produce anti-Müllerian hormones and androgens. These hormones induce MD regression and WD stability. The stabilized WDs subsequently differentiate into epididymides, vas deferens and seminal vesicles of the male reproductive tract. In the absence of these hormones in female embryos, the WDs regress while the MDs develop into the female reproductive tract (FRT) derivatives, i.e. the oviduct, uterine horns, cervix and the upper vagina (Roly et al., 2018). Prior to puberty, the vagina is composed of a solid epithelial cord derived from MD and UGS epithelia. The vagina becomes canalized in adulthood, resulting in the formation of the vaginal orifice at approximately 24-28 days, depending on the mouse strain (Kurita, 2010).

The mitogen-activated protein kinase kinase kinase 1 (MAP3K1) is a member of the MAP3K superfamily (Uhlik et al., 2004). The primary role of MAP3Ks is the regulation of mitogen-activated protein kinases (MAPKs), including extracellular signal-regulated kinases (ERKs), Jun N-terminal kinases (JNKs) and the p38 kinases. MAPKs, in turn, control diverse cell type-specific activities, such as gene expression, cell proliferation, migration, survival and death (Craig et al., 2008). MAP3K1 is a large protein consisting of 1500 amino acids with several well-defined functional domains, including the C-terminal kinase domain (KD), and the N-terminal guanine exchange factor (GEF), plant homeodomain (PHD) and armadillo repeat (ARM) domains (Wang et al., 2021a). These domains confer functional divergence to the protein. For example, the PHD domain possesses E3 ubiquitin ligase activity that mediates protein degradation, whereas the KD is responsible for stimulus-specific activation of the MAP2K–MAPK cascades (Lu et al., 2002; Xia et al., 1998).

Research in mice has shown that *Map3k1* gene mutations are associated with a plethora of developmental and physiological abnormalities in embryo survival, immune cell maturation and the development of sensory organs, such as eye and ear (Charlaftis et al., 2014; Gallagher et al., 2007; Labuda et al., 2006; Yousaf et al., 2015; Yujiri et al., 2000; Zhang et al., 2003). A connection between *MAP3K1* mutation and human diseases had not been established until recent genomic studies that associated gene mutations with sex differentiation defects (Das et al., 2013; Granados et al., 2017; Pearlman et al., 2010). Specifically, germline variants of *MAP3K1* in 13-18% of individuals with 46, XY disorder of sex development (DSD) have been found in clinical genetic data worldwide. The 46, XY DSD is defined as abnormal sexual differentiation in which patients with a male genotype are characterized by a female phenotype with respect to sexual features (Allen, 2009; Loke et al., 2014).

In light of these findings in humans, Warr et al. (2011) investigated the roles of MAP3K1 in male sexual development using *Map3k1^ΔKD^* mice. The *Map3k1^ΔKD^* genotype is a knockin/knockout strain in which the exon coding for the MAP3K1 KD is replaced by the gene *lacZ*, encoding β-galactosidase (β-GAL) (Xia et al., 2000). The resultant *Map3k1^ΔKD^* allele expresses a KD-deficient MAP3K1-β-GAL fusion protein. Through examination of the β-GAL activity in the *Map3k1^+/ΔKD^* embryos, MAP3K1 expression was found to be abundant in the gonads, testes, reproductive tracts and mesonephric tubules in E11-E13.5 embryos without sexual dimorphism. Although the expression pattern was consistent with the anticipated role of MAP3K1 in sex organ development, *Map3k1* homozygous knockout embryos did not exhibit major defects in testis development and the adult males were fertile. These results argue that MAP3K1 kinase activity is dispensable for male sexual development.

It is worth noting that the nature of gene mutations in mouse model and humans is fundamentally different. In the *Map3k1^ΔKD^* mice, an 800 bp deletion removes the KD coding sequences, resulting in a kinase-dead gene product. In contrast, in the patients with 46, XY DSD, the pathogenic nucleotide variants were spread throughout the GEF, PHD and ARM domains in the N-terminal coding regions (Ostrer, 2022). As the result, the mutant proteins have gain-of-function properties, resulting in increased binding of co-factors, i.e. RHOA and AXIN, and activation of downstream pathways, such as MAPKs and WNT (Chamberlin et al., 2019; Loke and Ostrer, 2012; Loke et al., 2014; Pearlman et al., 2010; Xue et al., 2019).

The human genomics data, indicating that MAP3K1 gain-of-function impedes male but potentiates female sexual features, led us to hypothesize that the loss-of-function protein would impede female sexual development. To test the hypothesis, we examined female sexual organ development in the *Map3k1^ΔKD^* mice. We showed that *Map3k1^ΔKD^* was autosomal recessive in causing developmental FRT abnormalities. Specifically, MAP3K1 KD deficiency impairs the caudal MD elongation and fusion with the UGS during development, likely through MAPK–WNT signaling crosstalk, leading to imperforate vagina and infertility in adult females. Our findings uncover a previously unreported genetic etiology and molecular mechanisms underlying FRT anomalies.

## RESULTS

### MAP3K1 KD deficiency impairs FRT formation and function

**Fig. 1.**
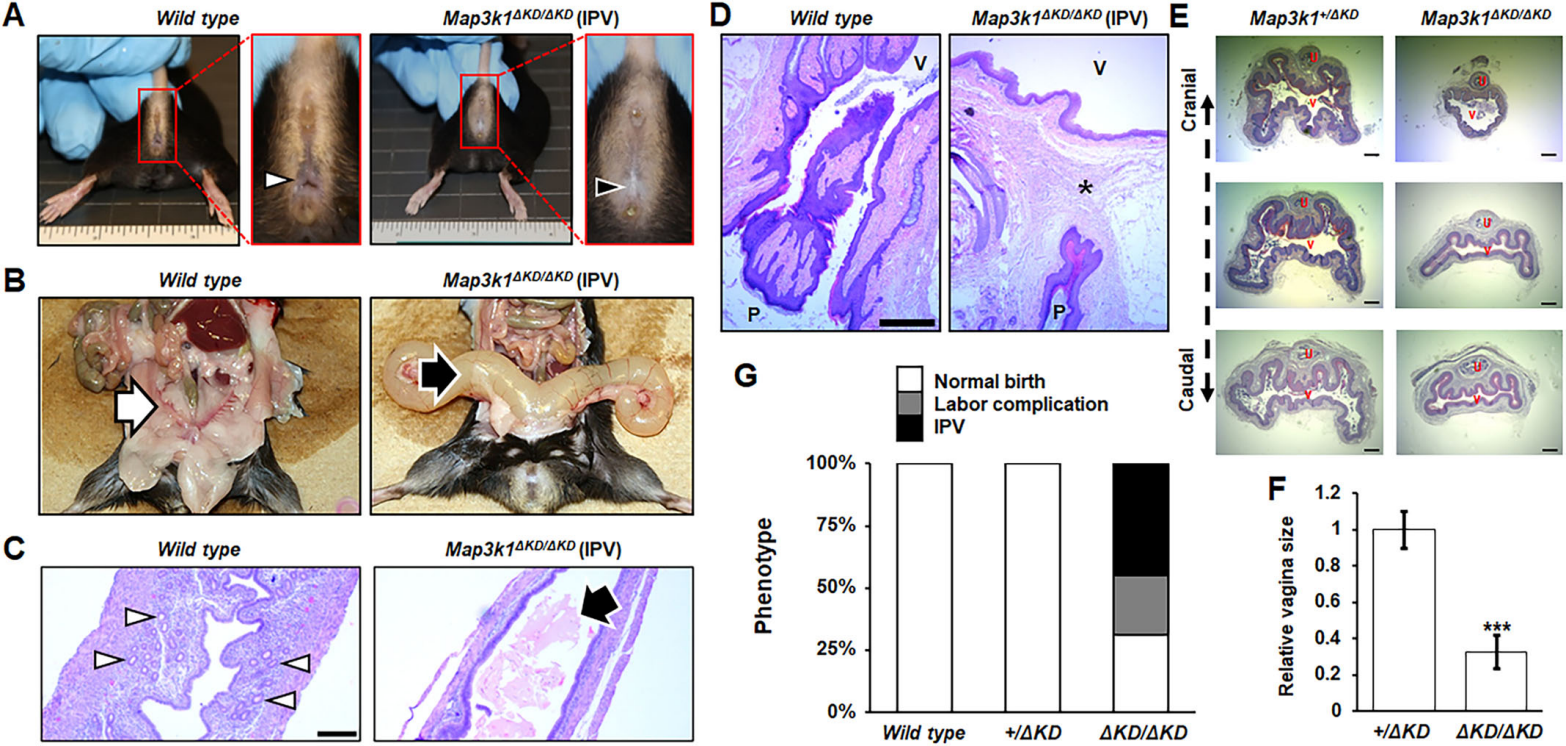
***Map3k1^ΔKD/ΔKD^* female mice have an imperforate vagina phenotype and reduced infertility.** Multiple 1- to 3-month-old wild-type and *Map3k1^ΔKD/ΔKD^* females were examined for reproductive defects. (A) Arrowheads point to the patent vaginal opening in wild-type females and its absence in the *Map3k1^ΔKD/ΔKD^* females with imperforate vagina (IPV). The ruler included for scale is in inches. (B) Arrows point to narrow uterine horns in wild-type females compared to dilated uterine horns in *Map3k1^ΔKD/ΔKD^* females with IPV. (C,D) Histology with Hematoxylin and Eosin (H&E) staining. (C) A narrow uterine lumen with a thick wall and prominent endometrial glands (arrowheads) in wild-type females was seen, compared to the dilated lumen containing proteinaceous secretory material admixed with exfoliated cellular debris (arrow), lined by a thinner uterine wall with fewer endometrial glands in *Map3k1^ΔKD/ΔKD^* females. (D) A canalized caudal vagina connected with the perineal skin in wild-type females was observed, in contrast to IPV with vaginal epithelium separated from the perineal skin by mesenchymal tissues (asterisk) in the *Map3k1^ΔKD/ΔKD^* females with IPV. (E,F) H&E staining images of the vaginal coronal sections (E) and calculation of the vagina size (F). Size of the vagina in the *Map3k1^ΔKD/ΔKD^* mice was compared to the position-matched size in *Map3k1^+/ΔKD^* mice. Values are mean±s.e.m. ***P<0.001 (two-tailed unpaired Student's *t*-test). (G) Quantification of female reproductive activity showed that although all of wild-type (*n*=70) and *Map3k1^+/ΔKD^* (*n*=97) mice had normal pregnancy and fertility, 77% of the *Map3k1^ΔKD/ΔKD^* mice (*n*=38) had abnormal reproduction due to either IPV or labor complications yielding no live pups. P, surface of perineal skin; U, urethra; V, vagina. Images are representative of at least three mice/genotype. Scale bars: 200 µm (C,E); 500 µm (D).

We examined the fertility of adult C57BL/6J mice with wild-type, *Map3k1^+/ΔKD^* and *Map3k1^ΔKD/ΔKD^* genotypes. As reported previously (Warr et al., 2011), the male *Map3k1^ΔKD/ΔKD^* mice displayed normal sexual organ development and fertility. In contrast, a significant proportion of the *Map3k1^ΔKD/ΔKD^* females were infertile due to the lack of a vaginal opening, a phenomenon known as an imperforate vagina (IPV) (Fig. 1A). Out of 38 *Map3k1^ΔKD/ΔKD^* females examined, 45% displayed IPV and, over time, developed lower abdominal distention. The swollen abdomen of the *Map3k1^ΔKD/ΔKD^* mice with IPV resulted from bilateral enlargement of the uterine horns containing protein-rich fluid, exfoliated keratin, leukocytes and necrotic epithelial cells (Fig. 1B,C). The medical designation for this condition is hydrometrocolpos. The uteri of wild-type mice displayed normal histology, two layers of myometrium, endometrium containing stromal and epithelial cells, and numerous epithelial-lined glands. In contrast, the uteri of *Map3k1^ΔKD/ΔKD^* mice with IPV displayed thin myometrial layers and minimal stromal cells in the endometrium, and lacked visible glands. Epithelial cells were also seen in the uterine lumen. Histological examination showed that the epithelium of the vagina and the surface of the perineal skin was continuous and outlined the canals of the caudal opening in wild-type mice; however, the epithelium was separated by the mesenchyme in the *Map3k1^ΔKD/ΔKD^* mice with IPV (Fig. 1D). Absence of the connecting epithelium prevented canalization at puberty and the mice with IPV could not be impregnated.

About 55% of *Map3k1^ΔKD/ΔKD^* females did not have the IPV phenotype; however, their lower FRT appeared to be narrowed by 60% compared to that of the heterozygous littermates (Fig. 1E,F). Consequently, although these knockout mice could be impregnated, half of them failed to deliver live offspring (Fig. 1G). Less than 30% knockout mice were fertile with no apparent reproductive defects, indicating a properly functioning hypothalamic gonadial axis. Neither the wild-type (*n*=70) nor the *Map3k1^+/ΔKD^* (*n*=97) mice had FRT defects or labor complications.

At puberty, the epithelial cord connecting the vagina and the UGS starts to canalize, resulting in vaginal opening. We examined this structure in sagittally sectioned FRTs by histology and immunohistochemistry using anti-keratin 14 (KRT14 or K14) for basal and anti-keratin 10 (KRT10 or K10) for suprabasal epithelial cells. At postnatal day (P) 21 prior to and after canalization, the vaginal epithelium in wild-type, *Map3k1^+/ΔKD^* and *Map3k1^ΔKD/ΔKD^* pups was K14 positive and K10 negative, whereas the UGS epithelium was positive for both K14 and K10. The epithelial cord, approximately 50 µm in length, connecting the two structures, was also K14/K10 double positive, similar to the UGS epithelium (Fig. S1A-D). One of the *Map3k1^ΔKD/ΔKD^* pups that did not have the epithelial cord had the vagina and UGS separated by ∼250 µm with the mesenchyme filling the space (Fig. S1E). As vaginal canalization cannot occur in the absence of the epithelial cord, this mouse would likely develop the IPV phenotype. These observations indicate that the *Map3k1^ΔKD^* allele is autosomal recessive in developmental abnormalities of the FRT.

### The developmental origins of the FRT defects

**Fig. 2.**
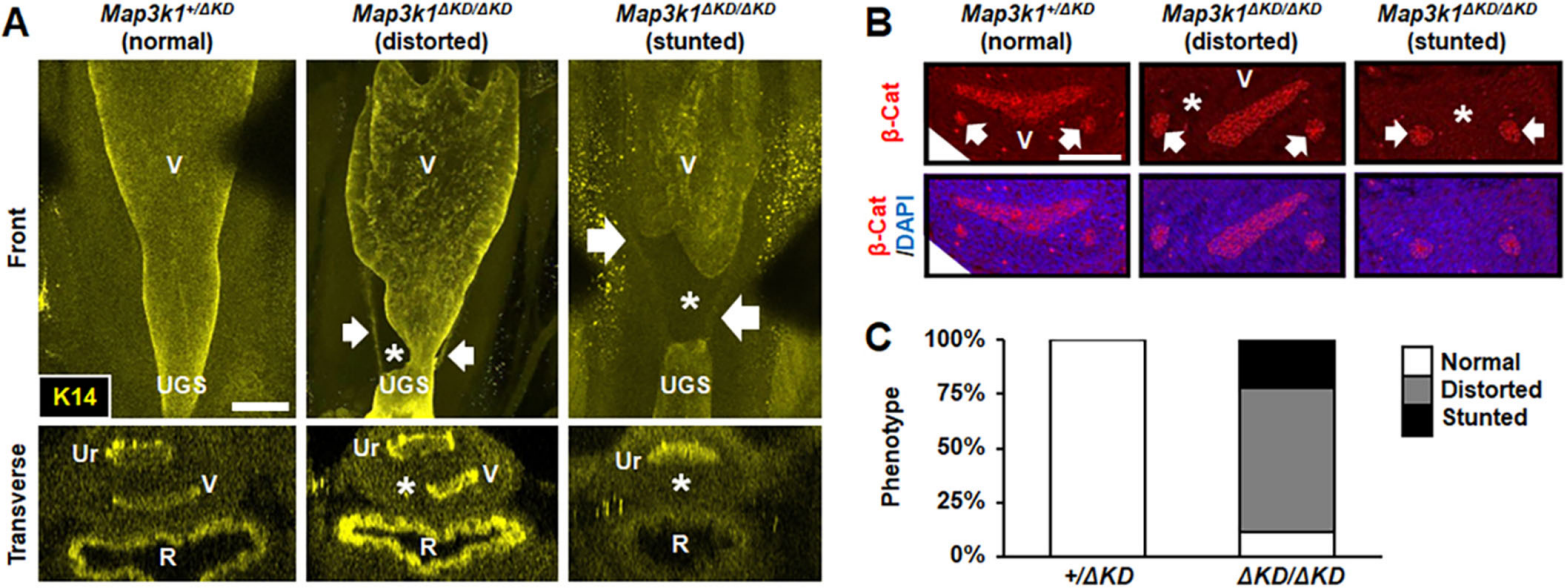
**Vagina–urogenital sinus fusion defects in *Map3k1^ΔKD/ΔKD^* neonates.** (A) The caudal end of the vagina and vagina–urogenital sinus (UGS) fusion site of *Map3k1^+/ΔKD^* (*n*=3) and *Map3k1^ΔKD/ΔKD^* (*n*=9) neonates (P0) were examined by whole-mount immunostaining with anti-keratin 14 (K14), which labels the epithelium lining the reproductive tract, and 3D images were captured with a confocal microscope. Representative longitudinal and transverse images are shown. (B) Transverse sections of the caudal reproductive tract of neonates were assessed by immunohistochemistry with anti-β-catenin (β-Cat) to label epithelial cells. Two representative defect phenotypes – distorted and stunted vaginas – observed in the *Map3k1^ΔKD/ΔKD^* mice are shown. (C) Distribution of the incidences of the normal, distorted and stunted vaginas found in *Map3k1^+/ΔKD^* and *Map3k1^ΔKD/ΔKD^* neonates. In A,B, arrows indicate the Wolffian ducts and asterisks mark the stunted and distorted vaginas in the *Map3k1^ΔKD/ΔKD^* pups. R, rectum; Ur, urethra; V, vagina. Images are representative of at least three mice/genotype. Scale bars: 200 µm (A); 100 µm (B).

We next asked whether the FRT abnormalities observed in *Map3k1^ΔKD/ΔKD^* adults were congenital through examination of the neonates, i.e. P0 mice. Using anti-K14 for whole-organ staining, we detected the expected symmetrical V-shaped vagina structure and vagina–UGS fusion zone in the *Map3k1^+/ΔKD^* females (Fig. 2A; Movies 1-3). In contrast, the vagina was largely distorted or absent due to truncated MDs failing to reach the UGS in the *Map3k1^ΔKD/ΔKD^* females. Immunohistochemistry using anti-β-catenin (CTNNB1), which labels epithelial cells, also detected a distorted or missing vagina in the caudal FRT of the *Map3k1^ΔKD/ΔKD^* pups, in contrast to the normal shape in the *Map3k1^+/ΔKD^* pups (Fig. 2B). Moreover, we found that eight out of nine (89%) *Map3k1^ΔKD/ΔKD^* pups had a distorted vagina structure (Fig. 2C), a ratio higher than that of infertile adults (70%), indicating that some, presumably modest, congenital defects can recover during postnatal development.

**Fig. 3.**
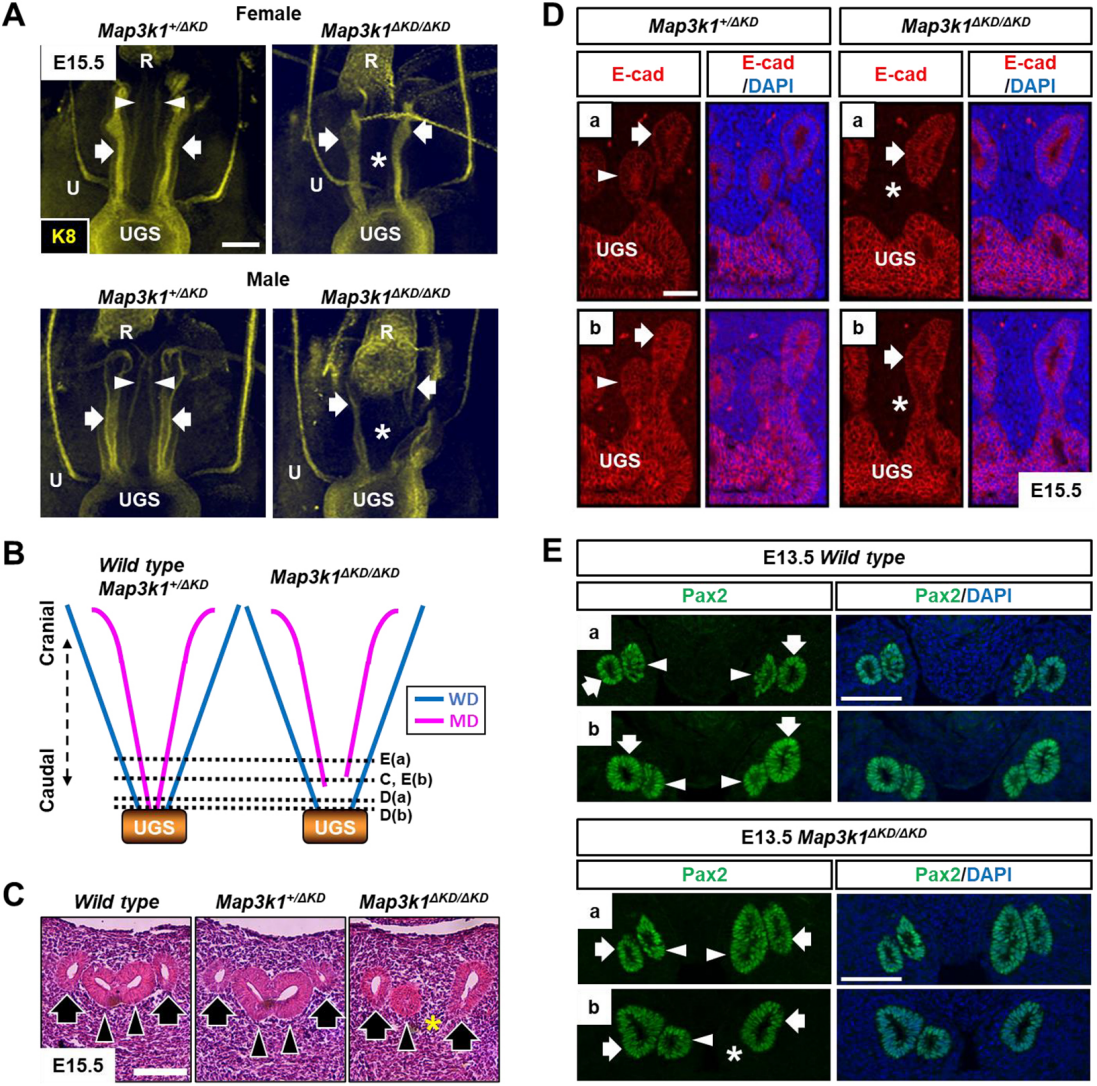
**MAP3K1 is required for Müllerian duct caudal elongation and establishment of Müllerian duct–urogenital sinus fusion.** (A) Reproductive tracts of female and male *Map3k1^+/ΔKD^* and *Map3k1^ΔKD/ΔKD^* E15.5 embryos were processed for whole-mount staining with anti-keratin 8 (K8), which labels the simple epithelia, and 3D images were captured with a confocal microscope. Representative longitudinal images of the developing urogenital system. (B) A diagrammatic illustration of the observed cranial and caudal reproductive tracts, where dotted lines mark the relative positions of the serial transverse sections shown in panels C-E. (C,D) The transverse sections at E15.5 were processed for H&E staining (C) and immunohistochemistry with anti-E-cadherin (E-cad) (D), a marker located on the epithelial cell membrane. (E) Transverse sections of the cranial and caudal reproductive tracts in wild-type and *Map3k1^ΔKD/ΔKD^* embryos at E13.5 were processed by immunohistochemistry to detect anti-Pax2, a marker of the developing reproductive tract epithelium. MD, Müllerian duct (arrowheads, red lines in B); WD, Wolffian duct (arrows, blue lines in B); R, rectum; U, ureter; UGS, urogenital sinus. Asterisks mark the absence of MDs in *Map3k1^ΔKD/ΔKD^* embryos. Images are representative of at least three mice/genotype. Scale bars: 100 µm (A,E); 50 µm (C,D).

To further trace the developmental origin of the FRT defects, we performed whole-mount immunostaining of E15.5 embryos using anti-keratin 8 (KRT8 or K8), a simple epithelial marker. The three-dimensional (3D) images showed that the WDs and the MDs extended and fused with the UGS in the expected manner in both female and male *Map3k1^+/ΔKD^* embryos (Roly et al., 2018) (Fig. 3A; Movies 4-7). The WDs also extended and fused with the UGS in the *Map3k1^ΔKD/ΔKD^* embryos, but the MDs were visibly truncated and MD–UGS fusion was abnormal or lacking. The caudal MD abnormality was observed in both female and male embryos.

To validate the caudal MD defects in *Map3k1^ΔKD/ΔKD^* embryos, we performed histological evaluation of transverse serial sections of the reproductive tract (Fig. 3B). Hematoxylin and Eosin (H&E) staining detected the expected two WDs and two MDs at the caudal sections of the reproductive tracts in wild-type and *Map3k1^+/ΔKD^* embryos (Fig. 3C). In contrast, there were two WDs but one MD in the corresponding sections of the *Map3k1^ΔKD/ΔKD^* embryos. Immunohistochemistry for E-cadherin (CDH1), which labels epithelial cells, confirmed these observations. Although the caudal end of the MD was fused with the UGS in *Map3k1^+/ΔKD^* embryos, it was disconnected from the UGS in the *Map3k1^ΔKD/ΔKD^* embryos (Fig. 3D). The MD defects were observed in all the 14 *Map3k1^ΔKD/ΔKD^* embryos but in none of the five wild-type and 19 *Map3k1^+/ΔKD^* embryos.

The defects observed in the E15.5 *Map3k1^ΔKD/ΔKD^* embryos could be due to insufficient proliferation or excessive apoptosis that led to breaking up of the already established MD–UGS connections. Cell proliferation [by 5-ethynyl-2′-deoxyuridine (EdU) labeling] and apoptosis [by terminal deoxynucleotidyl transferase dUTP nick end labeling (TUNEL) assay] were detected in the reproductive tracts of animals regardless of the genotype, and their levels were not quantitatively different in *Map3k1^+/ΔKD^* and *Map3k1^ΔKD/ΔKD^* embryos (Fig. S2A-D). These findings indicate that aberrant proliferation and apoptosis are not responsible for the MD defects in the knockout mice.

Alternatively, the defects could be due to a failure of MD caudal elongation and establishing the caudal MD–UGS connection. To test this possibility, we examined embryos at E13.5, a developmental stage when the MDs are just beginning to make contacts with the UGS (Mullen and Behringer, 2014). The serial transverse sections of the embryonic reproductive tract (positive for Pax2) revealed that the cranial MDs were identical in embryos of different genotypes, but the caudal MDs were strikingly different between wild-type and *Map3k1^ΔKD/ΔKD^* embryos (Fig. 3B,E). Although both MDs were caudally extended in the wild-type animals, either one or both of MDs were stunted in the *Map3k1^ΔKD/ΔKD^* embryos (Fig. 3E). These data suggest that MAP3K1 kinase activity is required for the caudal MD elongation to connect with the UGS.

### MAP3K1 regulates MAPK activity in the embryonic reproductive tract

**Fig. 4.**
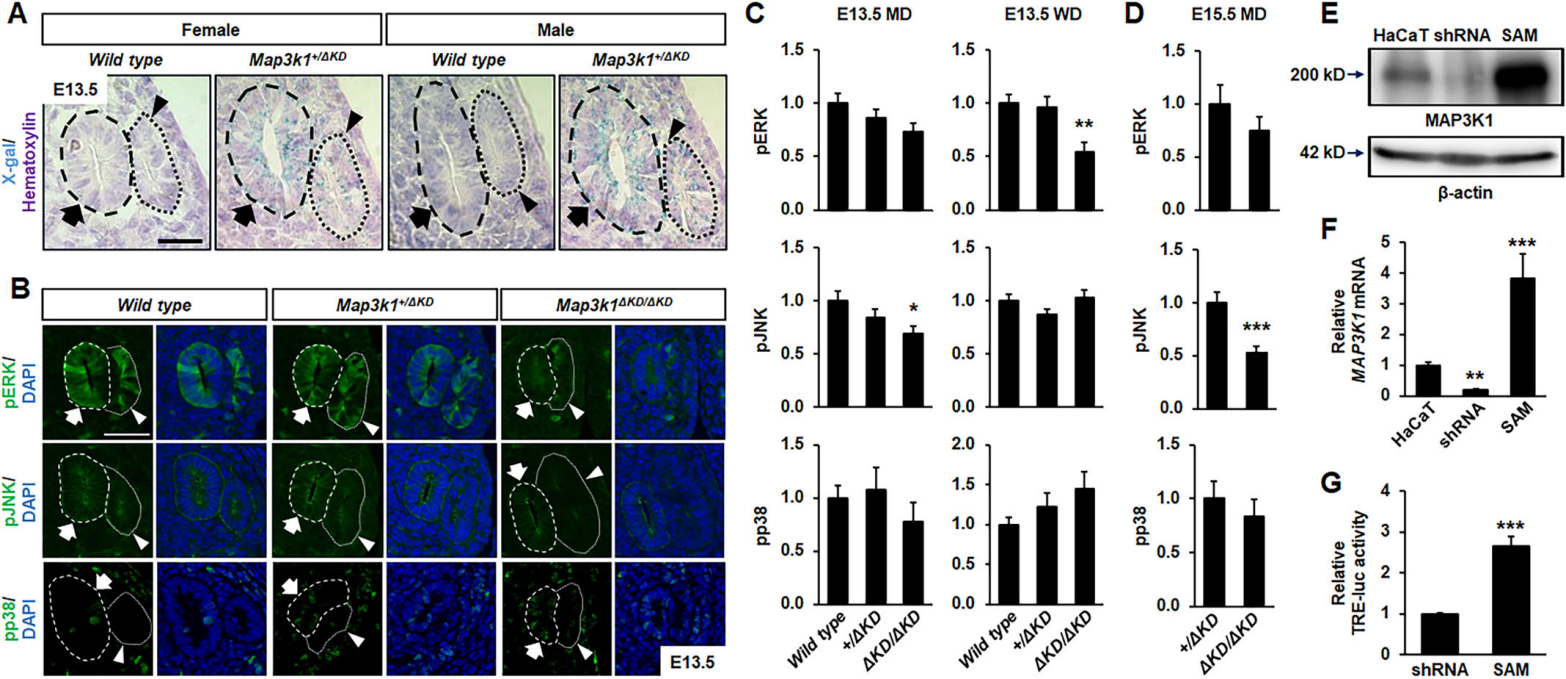
**MAP3K1 expression and signaling activity in the reproductive tract.** (A) Wild-type and *Map3k1^+/ΔKD^* embryos at E13.5 were processed for whole-mount X-gal staining. Representative sections were photographed with a bright-field light microscope. (B) Tissue sections of the reproductive tract from wild-type, *Map3k1^+/ΔKD^* and *Map3k1 ^ΔKD/ΔKD^* embryos were processed to immunolabel phosphorylated JNK (pJNK), ERK (pERK) or p38 (pp38). Representative images were captured using fluorescence microscopy. The WDs (arrows, dashed circles) and MDs (arrowheads, dotted circles) are marked. Scale bars: 50 μm (A,B). (C,D) Intensity of the fluorescence signal in the Müllerian ducts (MDs) and Wolffian ducts (WDs) was quantified at (C) E13.5 and (D) E15.5. At least three tissue sections/embryo and three embryos/genotype were examined at each time point. (E,F) HaCaT cells and genetically modified derivative cell lines (shRNA, for MAP3K1 knockdown, and SAM, for MAP3K1 overexpression) were validated by western blotting using anti-MAP3K1 (E) and qRT-PCR (F). Compared to the levels in HaCaT cells, MAP3K1 expression was significantly decreased in shRNA cells but significantly increased in SAM cells. (G) TRE-luc activities were determined after adjusting for the transfection efficiency with β-GAL. Statistical analyses of triplicate samples show significantly different TRE-luc activities. Values are mean±s.e.m. Values indicated by asterisks (*P<0.05, **P<0.01, ***P<0.001; one-way ANOVA followed by Tukey's multiple comparison test for C,F, two-tailed unpaired Student's *t*-test for D,G) are considered significantly different compared to values in wild type or *Map3k1^+/ΔKD^*, or to those in control cells (HaCaT or shRNA).

The *Map3k1^ΔKD^* allele encodes a MAP3K1-β-GAL fusion protein that is controlled by the endogenous *Map3k1* promoter (Xia et al., 2000). We performed whole-mount X-gal staining of E13.5 embryos to trace MAP3K1 expression. Compared to the lack of X-gal staining in the wild-type embryos, X-gal-positive cells (blue) were detected in the reproductive tract precursors in the *Map3k1^+/ΔKD^* embryos (Fig. 4A). The X-gal intensity was particularly strong on the luminal side of the WD and MD epithelium, although sporadic weak X-gal staining was also detectable in the surrounding mesenchyme. Thus, similar to its predominant epithelial expression we observed in other developmental tissues (Zhang et al., 2003), MAP3K1 is highly expressed in the epithelial tissue of the nascent reproductive tract.

To assess whether MAP3K1 KD deficiency affects MAPK activities, we examined MAPK phosphorylation in wild-type, *Map3k1^+/ΔKD^* and *Map3k1^ΔKD/ΔKD^* embryos. The phosphorylation of ERK and JNK was detected in the WD and MD epithelium, with the signal intensity particularly strong on the luminal side of the ducts (Fig. 4B). The phosphorylation of p38 MAPK (pp38) was virtually undetectable in the E13.5 embryos of all genotypes. Compared to that in wild-type and *Map3k1^+/ΔKD^* embryos, the phosphorylation of ERK (pERK) in *Map3k1^ΔKD/ΔKD^* embryos was significantly reduced in WDs (Fig. 4C). Phosphorylation of JNK (pJNK), in contrast, was found to be decreased in the MDs, but not the WDs, of the *Map3k1^ΔKD/ΔKD^* embryos. Similar observations were made for the *Map3k1^ΔKD/ΔKD^* E15.5 embryos in which the MDs displayed a significant reduction of pJNK (Fig. 4D).

To validate the role of MAP3K1 in MAPK activation in epithelial cells, we examined MAPK-dependent activation of AP-1 transcription factors that bind to the TPA-responsive element (TRE) to induce gene expression (Karin et al., 1997). We transfected the TRE-luciferase (TRE-luc) reporter into human epithelial HaCaT cells genetically modified to have increased MAP3K1 expression by the CRISPR/Cas9 synergistic activation mediator system (hereafter SAM cells) or decreased expression by small hairpin RNA knockdown (hereafter shRNA cells) (Fig. 4E). Compared to that in the parental HaCaT cells, the SAM cells had a nearly eightfold increase in MAP3K1 expression, whereas the shRNA cells had an 80% reduction (Fig. 4F). The TRE-luc activity, accordingly, exhibited a nearly threefold increase in SAM versus shRNA cells (Fig. 4G).

### MAP3K1 regulates morphogenesis and canonical WNT signaling

**Fig. 5.**
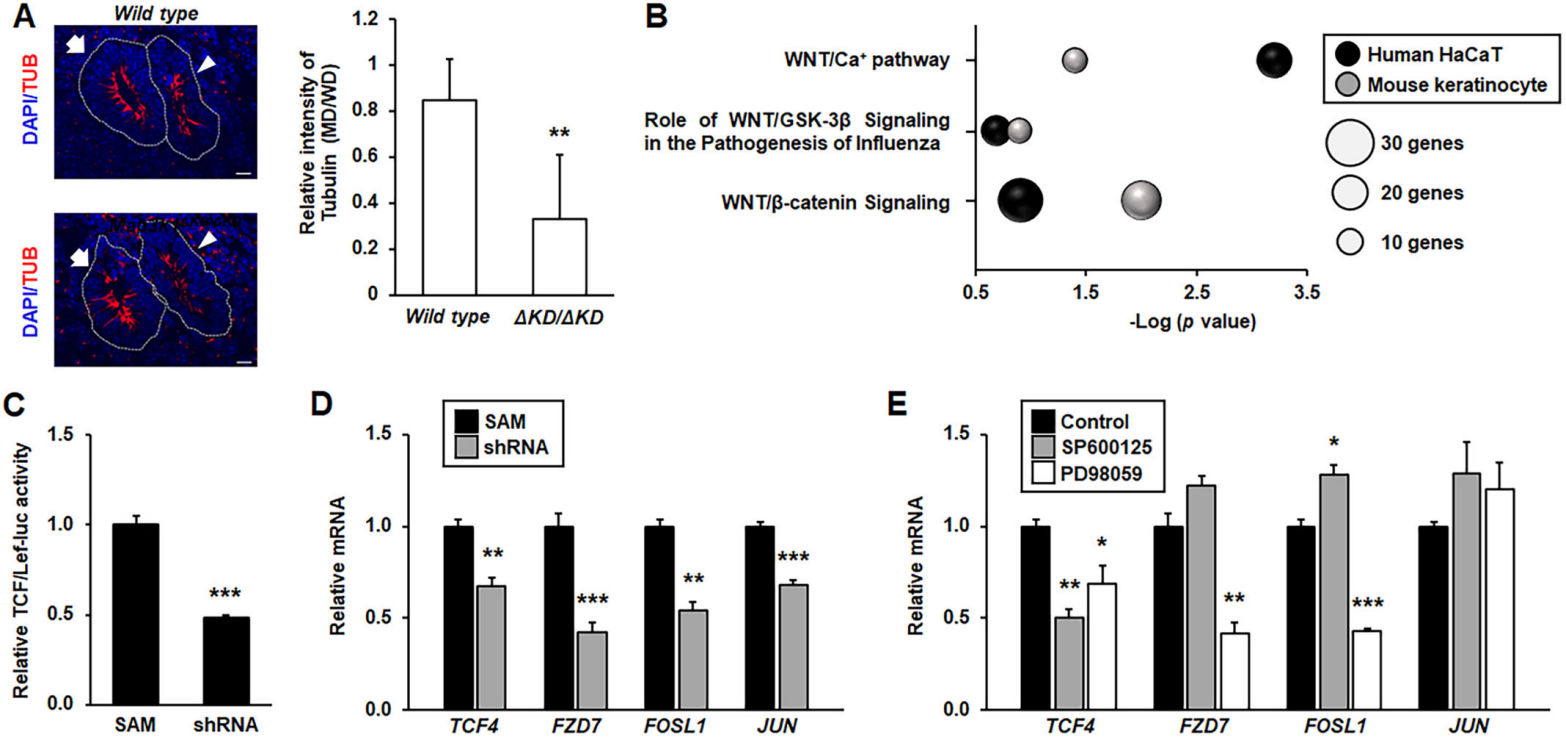
**MAP3K1 regulates WNT signaling through MAPK activity.** (A) Sections of female E13.5 wild-type (WT) and *Map3k1^ΔKD/ΔKD^* (KO) embryos were subjected to immunohistochemistry using anti-acetylated α-tubulin (TUB) for apical epithelial polarity (left). Scale bars: 50 μm. The signal intensity was measured and the levels in Müllerian ducts (MDs, arrowheads) were compared to those in Wolffian ducts (WDs, arrows) of the same genotype. (B) Ingenuity Pathway Analyses of differentially expressed genes in SAM HaCaT and shRNA HaCaT cells and in wild-type and *Map3k1^ΔKD/ΔKD^* mouse keratinocytes revealed that MAP3K1 deficiency downregulated WNT signaling. (C) Differential luciferase activities were detected in SAM and shRNA cells transiently transfected with TCF/Lef-luc (a WNT reporter construct). (D,E) Expression of candidate WNT target genes were examined by qRT-PCR in SAM and shRNA cells (D) and in SAM cells in the absence and presence of JNK (SP600125) and ERK (PD98059) inhibitors (E). Results represent data from at least three replicate experiments. Values are mean±s.e.m. Values indicated by asterisks (*P<0.05, **P<0.01, ***P<0.001; two-tailed unpaired Student's *t*-test for A,C,D, one-way ANOVA followed by Tukey's multiple comparison test for E) are significantly different between SAM and shRNA cells or control and inhibitor-treated samples.

The *in vivo* and *in vitro* data suggest that MAP3K1 regulates JNK/ERK activities, but how these pathways affect FRT development remains an open question. To address this question, we performed RNA sequencing (RNA-seq) using SAM and shRNA HaCaT cells, as well as keratinocytes derived from wild-type and *Map3k1^ΔKD/ΔKD^* mice (Wang et al., 2021b). A total of 3784 genes were differentially expressed by greater than twofold between shRNA and SAM cells, and 2825 genes were differentially expressed in wild-type and *Map3k1^ΔKD/ΔKD^* mouse keratinocytes. Enrichment analyses using Metascape software identified tissue morphogenesis and regulation of cell adhesion as the top MAP3K1-upregulated pathways (Fig. S3A,B). Interestingly, development of primary female sexual characteristics was found to be one of the top functions upregulated by MAP3K1 in HaCaT cells. To visualize the effect of MAP3K1 on morphogenesis, we measured the levels of acetylated α-tubulin, a marker of epithelial cell apical constriction and polarization for ductal morphogenesis (Andrew and Ewald, 2010; Booth et al., 2014; Fernandes et al., 2014; Xu et al., 2019). The acetylated α-tubulin was equally detected in the apical surface epithelium of WD and MD in the caudal reproductive tract of E13.5 wild-type embryos, and in the WD of the *Map3k1^ΔKD/ΔKD^* embryos (Fig. 5A). However, its detection was significantly reduced in the MD of *Map3k1^ΔKD/ΔKD^* embryos, suggesting that MAP3K1 has a MD-specific role in morphogenesis.

The RNA-seq data in MAP3K1-competent and -deficient cells also identified changes in WNT signaling. Ingenuity Pathway Analyses of the differentially expressed genes showed that MAP3K1 deficiency downregulated several WNT-related pathways, including the canonical WNT/β-catenin and the non-canonical WNT/Ca^2+^ pathways (Fig. 5B). When further validated using the canonical WNT pathway reporter, TCF/Lef-luc, we found that the luciferase activities in shRNA cells were slightly reduced by 50% compared to those in SAM cells (Fig. 5C). Moreover, several well-known canonical WNT signaling target genes, including *TCF4*, *FZD7*, *FOSL1* and *JUN*, were less expressed in shRNA than in SAM cells (Fig. 5D).

To assess whether the effects of MAP3K1 on canonical WNT signaling were mediated through the MAPKs, we treated the SAM cells with MAPK inhibitors and examined WNT target gene expression. Inhibition of JNK by SP600125 did not change the expression of *FZD7* and *JUN*, slightly increased the expression of *FOSL*, but significantly reduced the expression of *TCF4* (Fig. 5E). Inhibition of ERK by PD98059 had no effects on the expression of *JUN* and downregulated the expression of *TCF4*, *FOSL*, and *FZD7*. Our data indicate that MAP3K1 induces a moderate WNT activation, likely mediated in part through JNK and ERK.

### MAP3K1 activates WNT signaling via paracrine signaling

**Fig. 6.**
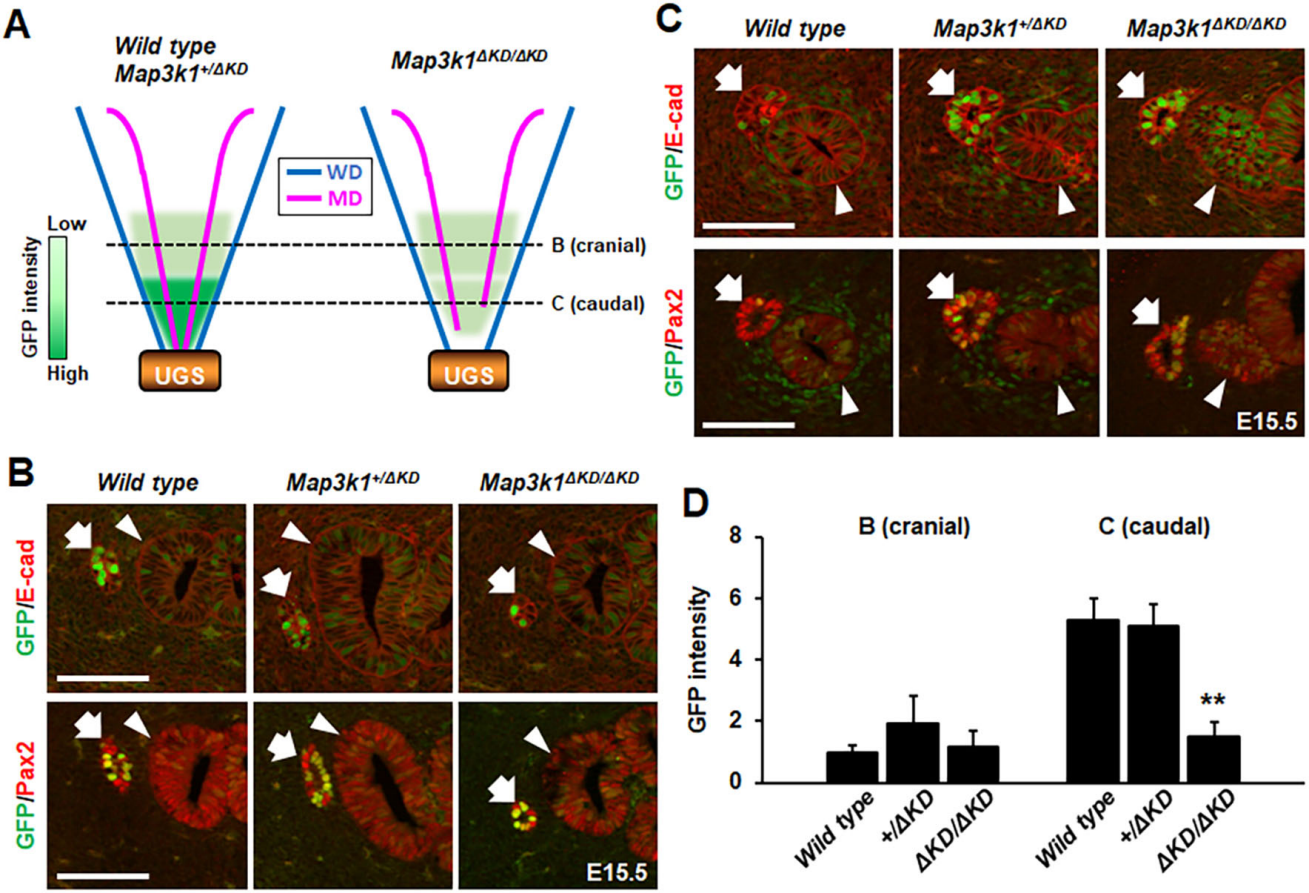
**MAP3K1 deficiency abolishes WNT signaling in the mesenchyme associated with the caudal reproductive tract.** The WNT reporter (TCF/Lef:H2B-GFP) was expressed in wild-type, *Map3k1^+/ΔKD^* and *Map3k1^ΔKD/ΔKD^* E15.5 embryos and these were processed to immunolabel Pax2 and E-cadherin. (A) Diagrammatic illustration of the observed GFP signal intensity in the cranial and caudal reproductive tract and the urogenital sinus (UGS). Dotted lines mark the relative positions of the transverse sections shown in panels B and C. (B,C) Representative images of cranial (B) and caudal (C) reproductive tracts were captured with a fluorescence microscope. Arrows, Wolffian ducts; arrowheads, Müllerian ducts. Scale bars: 100 μm (B,C). (D) GFP intensity in the mesenchyme of the cranial and caudal reproductive tracts was quantified. Values are mean±s.e.m. Values indicated by asterisks (**P<0.01; one-way ANOVA followed by Tukey's multiple comparison test) are considered significantly different from the wild-type embryo and the control media treatment.

The MAP3K1–WNT crosstalk *in vivo* was determined by introducing TCF/Lef:H2B-GFP in wild-type, *Map3k1^+/ΔKD^* and *Map3k1^ΔKD/ΔKD^* mice. The TCF/Lef:H2B-GFP links the gene for green fluorescent protein (GFP) with TCF/Lef, a WNT-inducible enhancer (Ferrer-Vaquer et al., 2010). We detected spatial WNT activities along the reproductive tracts of E15.5 embryos (Fig. 6). In the cranial reproductive tracts, there were evident GFP signals in the E-cadherin (epithelium) and Pax2 (developing reproductive tract epithelium) double-positive WD and MD epithelia, with little, if any, GFP positivity in the surrounding mesenchyme (Fig. 6A,B). There were no differences in GFP intensity among the *Map3k1* genotypes (Fig. 6D; Fig. S4A). In the caudal reproductive tract, by contrast, there was a distinct genotype-specific WNT activity. Although all embryos regardless of the genotypes had similar GFP signals in the WD and MD epithelia (Fig. S4B), only the wild-type and *Map3k1^+/ΔKD^* embryos, but not the *Map3k1^ΔKD/ΔKD^* embryos, had GFP positivity in the surrounding mesenchyme (Fig. 6A,C,D). Similar observations were made in E13.5 embryos, in which WNT activity in the caudal reproductive tract was significantly less in the mesenchyme, but not in the epithelium, of *Map3k1^ΔKD/ΔKD^* embryos than that in wild-type embryos (Fig. S4C-F).

**Fig. 7.**
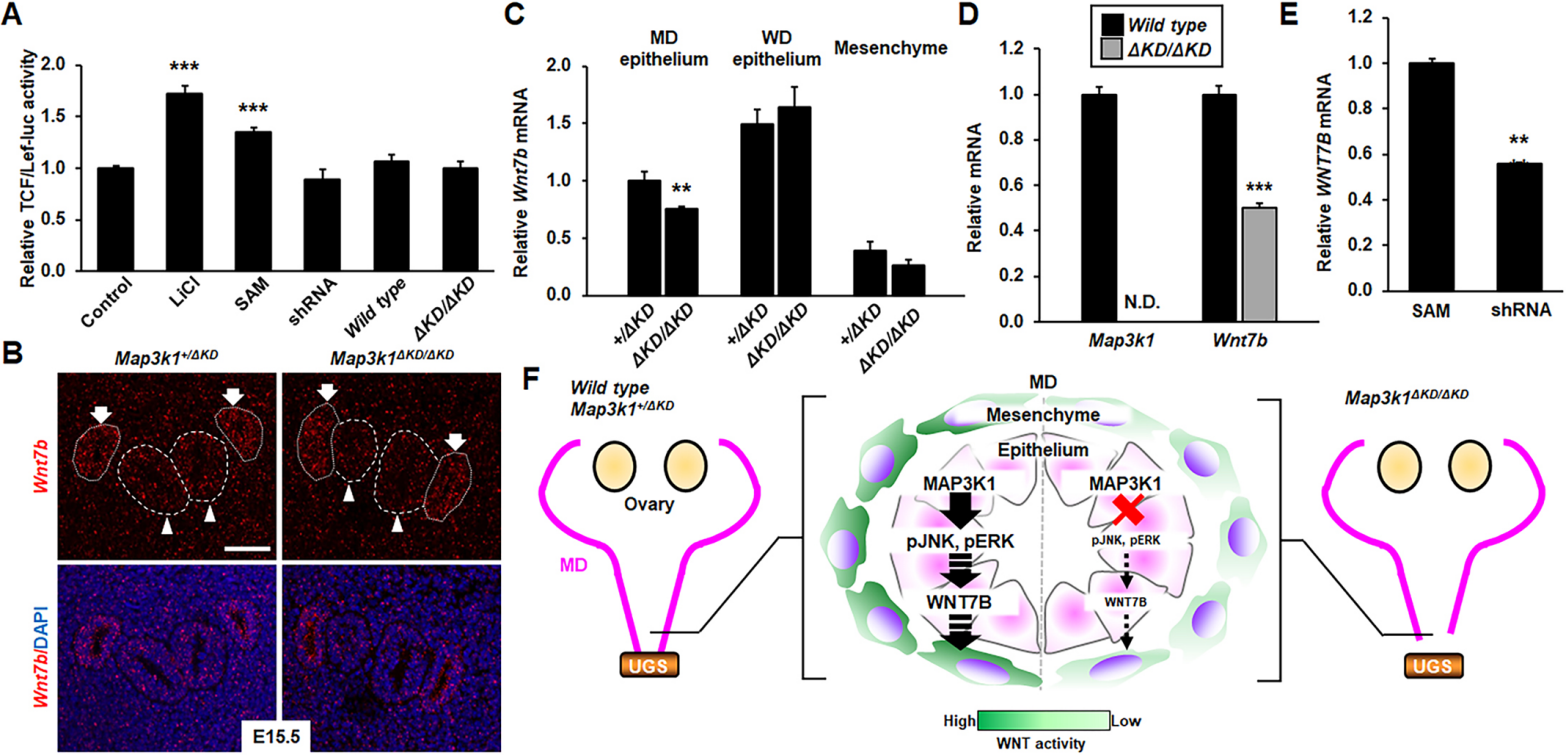
**MAP3K1 deficiency downregulates *Wnt7b* expression in the Müllerian duct epithelium.** (A) NIH3T3 cells transfected with TCF/Lef-luc and CMV-β-gal plasmids were treated with 20 mM LiCl or conditioned media from SAM-HaCaT or shRNA HaCaT cells, or from wild-type or *Map3k1^ΔKD/ΔKD^* MEFs. Relative luciferase activities normalized by β-GAL activites were calculated. (B) RNAscope *in situ* hybridization was performed using the *Wnt7b* probe on transverse sections of the reproductive tract. Representative images of *Wnt7b* expression in *Map3k1^+/ΔKD^* and *Map3k1^ΔKD/ΔKD^* female embryos at E15.5. Arrows, Wolffian ducts (WDs); arrowheads, Müllerian ducts (MDs). Scale bar: 50 μm. (C) Quantification of *Wnt7b* expression in the MD and WD epithelia and mesenchyme of *Map3k1^+/ΔKD^* and *Map3k1^ΔKD/ΔKD^* female embryos at E15.5. (D) The expression levels of *Map3k1* and *Wnt7b* mRNAs in wild-type and *Map3k1^ΔKD/ΔKD^* keratinocytes. Although the *Map3k1^ΔKD/ΔKD^* cells had no detectable *Map3k1* mRNA, they had significantly decreased expression of *Wnt7b*. N.D., not detected. (E) The level of *WNT7B* mRNA was lower in shRNA HaCaT cells than that in SAM cells. Values are mean±s.e.m. Values indicated by asterisks (**P<0.01, ***P<0.001; one-way ANOVA followed by Tukey's multiple comparison test for A, two-tailed unpaired Student's *t*-test for C-E) are considered significantly different from values in wild-type, *Map3k1^+/ΔKD^* and SAM cells. (F) Working model for the role of MAP3K1 in female reproductive tract development. MAP3K1 induces JNK/ERK phosphorylation and WNT7B expression in the MD epithelium to maintain WNT signaling in the mesenchyme for caudal MD elongation. MAP3K1 deficiency reduces mesenchymal WNT activity and causes defective MD caudal elongation and failure of MD–urogenital sinus (UGS) fusion.

As MAP3K1 was mainly expressed in the epithelium of the reproductive tracts, we speculated that the epithelial MAP3K1-regulated factors can transactivate WNT signaling in the surrounding MD mesenchyme. To test the paracrine mechanism, culture media from MAP3K1-competent and -deficient epithelial cells and mouse embryonic fibroblasts (MEFs) were collected and used to treat NIH3T3 fibroblasts transfected with the TCF/Lef-luc WNT reporter. Compared to the untreated cells, cells treated with lithium chloride (LiCl), a known canonical WNT activator, exhibited a twofold increase of luciferase activity. NIH3T3 cells treated with conditioned media collected from SAM HaCaT culture also had a significant 1.5-fold increase of luciferase activity; however, cells treated with media derived from shRNA HaCaT cells and from the wild-type and *Map3k1^ΔKD/ΔKD^* MEFs did not have such an increase (Fig. 7A).

One of the candidate paracrine activators are WNT ligands**,** known to be expressed in the internal genital epithelium and to play pivotal roles in FRT development (Mullen and Behringer, 2014). We examined WNT ligand expression in E15.5 *Map3k1^+/ΔKD^* and *Map3k1^ΔKD/ΔKD^* embryos using RNAscope *in situ* hybridization. We found that, in a MAP3K1-independent manner, *Wnt7a* was highly expressed in the MD epithelium, *Wnt5a* was expressed predominantly in the mesenchyme and *Wnt4* was expressed at low levels in both the epithelium and the mesenchyme of MDs and WDs (Fig. S5A-F). In contrast, *Wnt7b* expression was unaffected by MAP3K1 ablation in the WD epithelium and the surrounding mesenchyme but was significantly decreased in the *Map3k1^ΔKD/ΔKD^* MD epithelium (Fig. 7B,C). In cultured mouse keratinocytes, *Wnt7b* mRNA levels were reduced by 50% in *Map3k1^ΔKD/ΔKD^* cells compared to those in wild-type cells, and, in HaCaT cells, *MAP3K1* knockdown (shRNA) resulted in 40% reduction of *WNT7B* mRNA compared to cells in which MAP3K1 was overexpressed (SAM cells) (Fig. 7D,E).

## DISCUSSION

Human genomics data have associated *MAP3K1* mutations with 46, XY DSDs, in which the hyperactive MAP3K1 disrupts male sexual organ development (Loke et al., 2014; Pearlman et al., 2010). In this paper, we used a reverse genetics approach to explore whether hypo-activation of MAP3K1 affects the development of the opposite sex, i.e. females. We show that although male mice carrying kinase-inactive MAP3K1 have normal reproductive organs and fertility, female mice with the same kinase-inactive mutation exhibit defects of the reproductive tract. The defects originate in embryogenesis, during which the MDs in the *Map3k1^ΔKD/ΔKD^* embryos fail to extend caudally to connect with the UGS (Fig. 7F). This failure leads to distortion and complete absence of the MD–UGS junction in the neonatal homozygous knockout females, resulting in IPV, narrow lower reproductive tract, reduced fertility and labor complications in adults. Although all *Map3k1^ΔKD/ΔKD^* embryos have stunted MDs, some of the neonates display morphologically normal FRTs, and 30% of the adult females are fertile. These observations suggest that the MD abnormalities can be amended at later developmental stages; the mechanism for this restoration is currently unknown. Overall, the FRT defects in the *Map3k1^ΔKD/ΔKD^* mice complement the human genomics data and support a crucial role of MAP3K1 in sexual differentiation and sex organ development.

Studies in lymphoblastoid cells derived from patients with 46, XY DSD led to the proposition that MAP3K1 is upstream of MAPK and WNT signaling in sexual differentiation (Loke et al., 2014). Our data in the mouse models are consistent with this conclusion. Moreover, the availability of mouse embryos enabled us to further characterize the cell type-specific signaling activities of MAP3K1 in the developing sexual tissues. For example, MAP3K1 regulates distinct MAPKs in male and female reproductive tracts – it is required for optimal activation of ERK in the WDs but promotes the activation of JNK in the MDs. Additionally, although MAP3K1 deficiency did not alter the canonical WNT activity in the cranial reproductive tract, it reduced the activity in the caudal reproductive tract. More specifically, the *Map3k1*-null caudal reproductive tract displayed reduced WNT activity in the mesenchyme, but not in the epithelium. In this context, it is interesting to note that the mesenchyme-specific β-catenin knockout causes stunted FRT, reminiscent of the phenotype resulting from *Map3k1* deficiency (Deutscher and Hung-Chang Yao, 2007; MacDonald et al., 2009). Results in the mouse model reveal a considerable complexity of MAP3K1 signaling *in vivo*, likely the results of temporal-spatial morphogenetic signals and their dynamic crosstalks in the developing tissues.

To explain how abundant MAP3K1 expression in epithelium of the reproductive tracts leads to canonical WNT activity in the surrounding mesenchyme, we tested the idea that MAP3K1 regulates WNT ligand expression for paracrine WNT activation. Lending support to the idea are the findings that conditioned media from MAP3K1-competent, but not -deficient, epithelial cells activated the TCF/Lef-luc in fibroblasts. Several WNT ligands, i.e. WNT4, WNT7A and WNT5A, are known to be expressed in the reproductive tract and play a crucial role in FRT morphogenesis (Mericskay et al., 2004; Miller and Sassoon, 1998; Parr and McMahon, 1998; Prunskaite-Hyyrylainen et al., 2016; Vainio et al., 1999); however, their expression turned out to be independent of MAP3K1. In contrast, we found that expression of WNT7B, a ligand known to be expressed in the FRT epithelium (Seishima et al., 2019), was reduced in the MAP3K1-deficient MD epithelium and cultured epithelial cells. WNT7B is implicated in the regulation of epithelial morphogenesis through a mechanism involving epithelial–mesenchymal interaction (Kispert et al., 1998), but its roles in sexual tissue development have not been established in mice, because *Wnt7b* homozygous knockout mice die before E11.5 owing to placental abnormality and the heterozygotes have normal development similar to the wild-type mice. There are other strong candidates of WNT ligands, such as WNT9B, which is produced from the WD epithelium to trans-regulate MD caudal elongation (Roly et al., 2018). Unfortunately, we were unable to examine these owing to unavailability of validated RNA probes. We believe that future studies of MAP3K1-dependent transcriptomes in the caudal reproductive tract would facilitate a better understanding of additional WNT ligands and other molecular factors that mediate the MAP3K1–WNT crosstalk.

Global gene expression data in MAP3K1-competent and -deficient epithelial cells has shown that MAP3K1 deficiency prevents the optimal activation of not only the canonical, but also the non-canonical WNT pathways. Non-canonical WNT signaling is a key regulator for the establishment of cell polarity in positional cell movement, morphogenesis and ductal organ formation (Karner et al., 2006; Shi, 2022; Sugimura and Li, 2010). In line with the pathway analyses data, we found that MAP3K1 deficiency reduces acetylated α-tubulin, a fundamental marker for cell polarization, in the apical surface of the MD epithelium (Andrew and Ewald, 2010; Xu et al., 2019). In this context, a potential interaction partner of MAP3K1 in sexual differentiation is RHOA, known to act in the non-canonical WNT pathway and regulate cell polarity (Fanto et al., 2000; Kim and Han, 2005). We have additionally noted in this work that MAP3K1 kinase deficiency causes an approximate 50% reduction of the downstream signaling and variable severity of the FRT defects. It is thus reasonable to speculate the existence of functionally redundant molecules that partially compensate for the loss of MAP3K1 activity. For example, the MAP3K1 interaction partners, including RHOA, MAP3K4 and AXIN2, could potentially modify its activity in sexual development, an idea yet to be tested genetically (Loke et al., 2014).

Aberrant FRT development in humans results in dysplasia or absence of the vagina, cervix and/or caudal uterus. These conditions affect approximately 5% of human females and are difficult to diagnose (Chan et al., 2011; Choussein et al., 2017). Clinical symptoms include, but are not limited to, failure to menstruate, periodic lower abdominal pain and a range of anomalies that affect multiple organs in the abdomen (Bombard and Mousa, 2014). Patients with this condition are at a greater risk of adverse reproductive outcomes, including reduced fertility, an increased miscarriage rate, preterm delivery, fetal malpresentation and complications at delivery (Chan et al., 2011). Research using genetic mouse models has identified genes that play pivotal roles in MD development (Parr and McMahon, 1998; Prunskaite-Hyyrylainen et al., 2016). Some of these genes, such as *Wnt4* and *Hoxa10*, are found to be mutated in humans with FRT anomaly, underscoring conserved genetic control of FRT development across the species (Biason-Lauber et al., 2004; Cheng et al., 2011). To date, the molecular mechanisms underlying MD development remain poorly understood and the molecular etiology for the majority of the anomalous cases is yet to be identified (Laronda et al., 2013). Our findings indicate that MAP3K1 loss of function is a molecular etiology contributing to congenital female reproductive defects in mice that is likely conserved across species.

## MATERIALS AND METHODS

### Mice

Animals were housed in a pathogen-free vivarium in accordance with institutional policies. All animal experiments were approved in advance by the Institutional Animal Care and Use Committee at the University of Cincinnati College of Medicine. The *Map3k1^ΔKD^* mice were generated in house and described previously (Xia et al., 2000; Zhang et al., 2003). The Tg(TCF/Lef1-HIST1H2BB/EGFP)61Hadj (TCF/Lef:H2B-GFP) mice were from The Jackson Laboratory (JAX strain 013752) (Ferrer-Vaquer et al., 2010). The mice were backcrossed with C57BL/6J for more than 12 generations to generate B6 congenic background. Tail genomic DNA was used for PCR genotyping.

### Cells, reagents and antibodies

HaCaT cells, a spontaneously immortalized human keratinocyte line, and NIH3T3 fibroblasts were obtained from American Type Culture Collection (ATCC). The wild-type and *Map3k1^ΔKD/ΔKD^* mouse keratinocytes and mouse embryonic fibroblasts (MEF) were prepared as described previously (Wang et al., 2021b; Zhang et al., 2003). The mouse keratinocytes were maintained in defined keratinocyte serum-free medium without calcium (K-SFM without calcium, Gibco) and routinely sub-cultured. The HaCaT cells, MEFs and NIH3T3 cells were cultured in Dulbecco's modified Eagle's medium (DMEM); the cell culture supplements added to DMEM, including fetal bovine serum (FBS), glutamine, nonessential amino acids, penicillin and streptomycin, were from Gibco. All other reagents and their sources are summarized in Table S1; antibodies are listed in Table S2.

### Modulating MAP3K1 expression in HaCaT cells

The *MAP3K1* shRNA lentiviral vectors were purchased from Sigma-Aldrich (Clone ID, TRCN000000616) and plasmids for the CRISPR/Cas9 SAM system, i.e. lenti-sgRNA(MS2)_puro, dCas9VP64 and MS2-P65-HSF1, were purchased from Addgene (#73797, #61425 and #61426). The single guide RNAs (sgRNAs) for *MAP3K1* were designed based on publicly available filtering tools (https://portals.broadinstitute.org/gpp/public/analysis-tools/sgrna-design) (Table S3); the sgRNA was cloned into lenti-sgRNA(MS2)_puro. The lentiviral vectors were co-transfected with the packaging psPAX2 (Addgene #12260) and envelope pMD2.G (Addgene #12259) plasmids into 293T packaging cells (ATCC). Viral production, amplification and titration were done following the protocol in Addgene (https://www.addgene.org/protocols/aav-production-hek293-cells/).

To make MAP3K1-deficient HaCaT cells, the cells were transduced with lentivirus containing *MAP3K1* shRNA and then selected with puromycin to obtain stable ‘shRNA’ cells. To make MAP3K1 overexpressing HaCaT cells, the SAM system was used (Konermann et al., 2015). Specifically, the lentiviruses for sgRNAs, dCas9VP64 and MS2-P65-HSF1 were used to transduce the HaCaT cells, which were subsequently selected with 3 mg/ml puromycin, 10 mg/ml blasticidin and 5 mU hygromycin in DMEM containing 5% FBS to generate the stable ‘SAM’ cells. The SAM cells were treated with 10 µM SP600125 (JNK inhibitor; Calbiochem, La Jolla, CA, USA), 10 μM PD98059 (ERK inhibitor; Cell Signaling Technology, Danvers, MA, USA) or DMSO as vehicle control for 24 h before isolation of RNA.

### Histology, immunohistochemistry and RNAscope *in situ* hybridization

The lower bodies of appropriately euthanized embryos and neonates were collected and fixed by immersion in 4% paraformaldehyde (PFA, Thermo Fisher Scientific) overnight at 4°C. For microscopic analyses, the fixed tissues were embedded routinely in paraffin, sectioned serially at 5 µm using a rotary microtome (MT-980, Research & Manufacturing Co., Inc, Tucson, AZ, USA), and stained with conventional H&E. Images were captured by bright-field light microscopy with different magnifications.

For immunohistochemistry, fixed tissues were immersed with sucrose gradients (15% and 30%), covered in Tissue-Tek OCT Compound, flash frozen on dry ice and stored at −80°C. Serial 12-μm-thick sections were obtained and washed in PBS, immersed in boiled 10 mM citrate buffer (pH 6.0) for 30 min, blocked with PBS containing 5% bovine serum albumin (BSA) for 1 h at room temperature (RT), and incubated with a diluted primary antibody (Table S2) overnight at 4°C, followed by secondary antibody incubation and Hoechst 33342 staining.

RNAscope *in situ* hybridization was done as described previously (Wang et al., 2012) and following the manufacturer's instructions. Briefly, 12-µm-thick sections were deparaffinized and washed. After epitope retrieval in boiled citrate buffer (10 mM, pH 6.0) for 30 min, the sections were incubated with the commercial synthetic probes (Table S1) at 40°C, followed by signal amplification and fluorescent dye labeling. Images were captured by the Zeiss Axio fluorescence microscope.

Microscopic evaluation of the adult FRT was done at the Comparative Pathology and Mouse Phenotyping Shared Resource of the Ohio State University College of Veterinary Medicine. At the necropsy of 1- to 3-month-old female mice, animals were appropriately euthanized, and the reproductive tract and selected major viscera were removed and fixed by immersion in neutral buffered 10% formalin. The samples were processed routinely into paraffin, sectioned at 5 µm, stained with H&E, and evaluated by American College of Veterinary Pathologists board-certified veterinary pathologists.

### Whole-mount tissue immunofluorescence staining and X-gal staining

A previously described protocol was adapted for whole-organ immunofluorescence (Messal et al., 2021). Briefly, the lower bodies of embryos and neonates were fixed with 4% PFA overnight at 4°C. For antigen retrieval, the tissues were incubated with FLASH reagent 2 (200 mM borate buffer, 250 g/l urea and 80 g/l Zwittergent) for 1 h at RT, followed with overnight incubation in FLASH reagent 2 at 55°C. For immunostaining, the tissues were washed with PBS containing 0.2% Triton X-100 (PBST) for 30 min at RT, immersed in blocking buffer [PBST with 10% (wt/vol) FBS, 0.02% (wt/vol) sodium azide, 1% (wt/vol) BSA and 5% (vol/vol) DMSO] for 1 h at RT, and subjected to immunolabelling with primary and secondary antibodies (Table S2) for 5 and 3 days, respectively, at RT. For tissue clearing, after extensive washing, tissues were dehydrated gradually in 30%, 50%, 75% and 100% (vol/vol) methanol, followed by a serial gradient with 25-100% of benzyl alcohol/benzyl benzoate (BABB). Cleared tissues were placed in chamber slides and imaged using a Zeiss confocal microscope (LSM700).

For whole-mount X-gal staining, the lower bodies of wild-type and *Map3k1^+/ΔKD^* embryos were collected and fixed with a solution containing 2% PFA, 0.2% glutaraldehyde, 5 mM EGTA, 2 mM MgCl_2_ and 0.02% NP40 for 20 min at RT. The tissues were immersed in staining solution, containing 100 mM phosphate buffer, 5 mM K_3_Fe(CN)_6_, 5 mM K_4_Fe(CN)_6_, 2 mM MgCl_2_, 0.02% NP40 and 1 mg/ml X-gal overnight at 37°C. The tissues were post fixed with 4% PFA at 4°C overnight, processed through sucrose gradient, embedded in OCT, flash frozen, and sectioned at 12 µm. The sections were counterstained with diluted Hematoxylin for 5 min at RT. After dehydration through ethanol and xylene, sections were covered in mounting medium xylene, and images were captured under a bright-field light microscope with different lenses.

### Proliferation and apoptosis detection

Embryos were harvested from pregnant mice given an intraperitoneal injection of EdU (5 mg/kg body weight) 2 h prior to collection. The embryos were fixed, embedded and sectioned as described above for immunohistochemistry. Cell proliferation was determined with the iClick EdU Imaging Kit (ABP Biosciences) and apoptosis was measured using the ApopTag Peroxidase *In Situ* Apoptosis Detection Kit (Millipore Sigma), following the manufacturers' protocols. Images were captured with a Zeiss Axio microscope. The positively stained cells were quantified from at least three embryos/genotype in four sections per embryo encompassing the lower reproductive tract.

### Transfection and reporter assays

Transfection of the NIH3T3, HaCaT SAM and shRNA cells was performed using Lipofectamine 2000 (Invitrogen). Briefly, cells were seeded in 24-well tissue culture plates and transfected with TRE-luc (Addgene, #16535) and TCF/Lef-luc (Addgene, #16558) plasmids, together with the β-galactosidase plasmid (Addgene, #16486), following the manufacturer's instructions.

Serum-free DMEM incubated for 24 h with shRNA or SAM HaCaT cells, or wild-type or *Map3k1^ΔKD/ΔKD^* MEFs was collected as ‘conditioned media’. The *in vitro* studies of epithelial–mesenchymal interaction were performed by measuring TCF/Lef-luc activity in transfected NIH3T3 cells. The transfected cells were treated with the conditioned media for 24 h, and cell lysates were examined for luciferase activities with the luciferase assay system kit (Promega). The relative luciferase activities were calculated after normalization with the β-GAL activities. Results represent duplicate data from at least three biological samples of each genotype.

### RNA isolation and reverse transcription and real-time PCR

Total RNA was extracted from cultured cells and purified with PureLink RNA Mini kit (Invitrogen). The RNA (0.5 μg) was subjected to reverse transcription using the SuperScript IV Reverse Transcriptase kit (Invitrogen). The cDNA was subjected to quantitative real-time PCR (qRT-PCR) using the MX3000p thermal cycler system (Agilent Technologies, Santa Clara, CA, USA) and SYBR Green qPCR Master Mix (Applied Biosystems, Waltham, MA, USA). Relative gene expression was calculated by the comparative ΔΔCt method normalized to a constitutively expressed housekeeping gene (*GADPH*). All determinations were performed with triplicate samples. Primer sequences are listed in Table S4.

### RNA-seq and pathway analyses

Total RNA was isolated from SAM and shRNA HaCaT cells. The RNA quality was analyzed by Bioanalyzer (Agilent Technologies). RNA sequencing was performed with biological triplicate samples by the Genomics, Epigenomics and Sequencing Core at the University of Cincinnati using established protocols described previously (Wang et al., 2022). Briefly, Poly(A) RNA was isolated from 1 μg total RNA using the NEBNext Poly(A) mRNA magnetic isolation module (New England Biolabs, Ipswich, MA, USA) and enriched with SMARTer Apollo Automated NGS Library Prep System (Takara Bio USA, Mountain View, CA). The cDNA library was made using the NEBNext Ultra II Directional RNA Library Prep kit (New England BioLabs) with a PCR cycle number of 8, followed by library quality control and quantification via qRT-PCR (NEBNext Library Quant Kit, New England Biolabs). The individually indexed libraries were proportionally pooled and loaded on a NextSeq 550 sequencer (Illumina, San Diego, CA, USA) under the sequencing setting of single read 1×85 bp. Once sequencing was completed, adapter-trimmed FASTQ files for downstream data analyses were generated via the Illumina BaseSpace Sequence Hub. A total of 23.4±1.6 (mean±s.e.m.) million pass filter reads per sample were generated. For each sample, >97% reads were aligned to the MM10 reference genome, including >99% of stranded sequences, and ∼85% reads aligned to the coding and untranslated regions, which indicated good RNA and data quality. General bioinformatics were performed using the BaseSpace app, RNA-Seq Alignment app v2.0.2, with ‘Mark duplicates’ unchecked, followed by the RNA-seq Differential Expression app version 1.0.1. The analyses used STAR (http://code.google.com/p/rna-star/) for alignment and Salmon (https://github.com/COMBINE-lab/salmon) for quantification (transcripts per million), followed by DESeq2 (Love et al., 2014) to identify differentially expressed genes. Significant genes were selected based on adjusted *P*-value <0.05. The differentially expressed genes were subjected to Bioinformatics analyses using Ingenuity Pathway Analyses software (IPA, Qiagen) and Metascape (Zhou et al., 2019). Similar bioinformatics analyses were performed using RNA-seq data derived from wild-type and *Map3k1* knockout mouse keratinocytes as we have done previously (Wang et al., 2021b).

### Image quantification and statistical analyses

The ratio of EdU- or TUNEL-positive cells versus the total epithelial cells in MDs and WDs was calculated. The immunofluorescence intensity (i.e. by immunohistochemistry, GFP and RNAscope) was measured in the MD and WD epithelia and the surrounding mesenchyme. The WD and MD areas consisting of epithelial cells expressing Pax2, E-cadherin, pJNK, pERK, pp38, acetylated α-tubulin and Hoechst 33342 were outlined, and the mean intensity values of target proteins/genes were measured. Quantification was done by ImageJ software (version Fiji-2.1.1). The level of the targets in the images was determined after background subtraction. At least three embryos/genotype were used for quantification.

All data are shown as mean±s.e.m. Statistical comparisons were performed with two-tailed unpaired Student's *t*-test or one-way ANOVA followed by Tukey's multiple comparison test. *P<0.05, **P<0.01 and ***P<0.001 were considered to be statistically significant.

## Supplementary Material

10.1242/dmm.050669_sup1 Supplementary information
