# Supplementary material for: MAP3K1 regulates female reproductive tract development
Source: Dis Model Mech. 2024 Mar 28;17(3):dmm050669. doi: 10.1242/dmm.050669 (PMC10985838; doi:10.1242/dmm.050669)
Supplement: Supplementary information [file dmm-17-050669-s1.pdf]

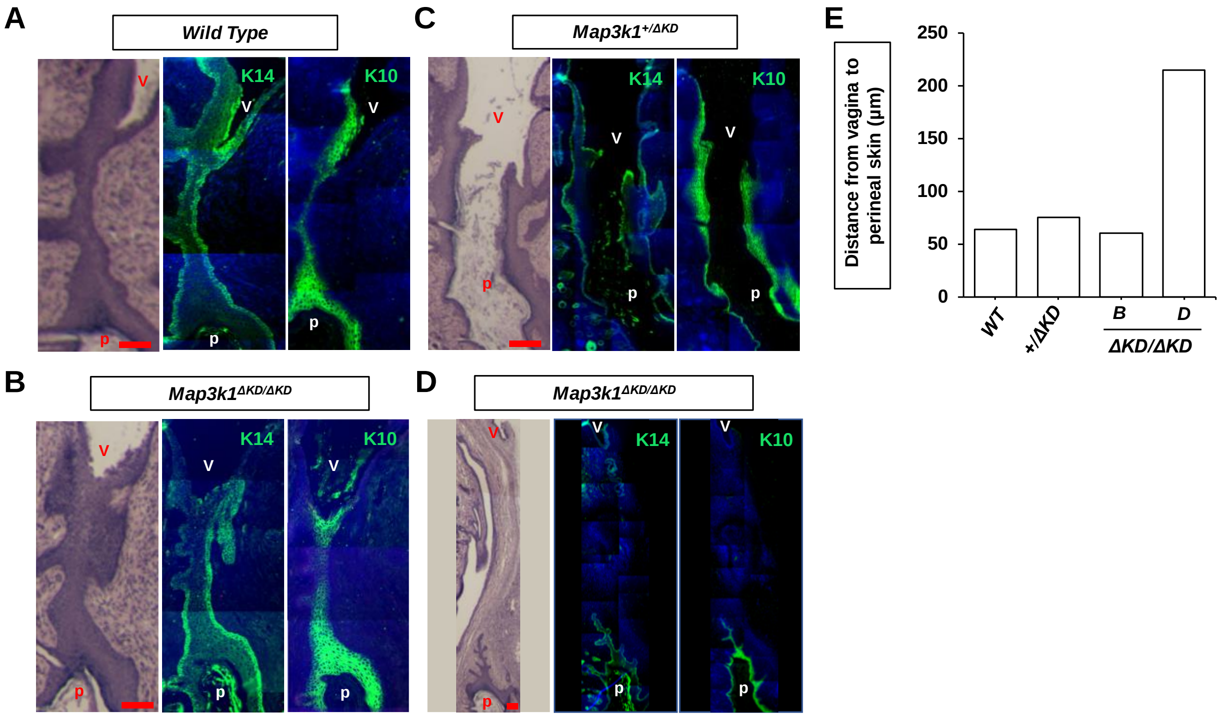

Fig. S1. Characterization of the vagina structure at puberty. Sagittal sections of the reproductive tracts in female P21 mice were examined by H&E staining and immunohistochemistry using anti-K14 and anti-K1. (A and B) Pre-canalization of the FRT in wild type and *Map3k1*$^{\Delta KD/\Delta KD}$ mice and (C) post-canalized FRT of a *Map3k1*$^{+/\Delta KD}$ mouse and (D) the FRT of a *Map3k1*$^{\Delta KD/\Delta KD}$ mouse lacking the epithelial cord between vagina and UGS. (E) The distance between vagina and UGS was measured in 4 samples. V, vagina, P, surface of perineal skin. Scale bars, 5 µm.

Disease Models & Mechanisms • Supplementary information

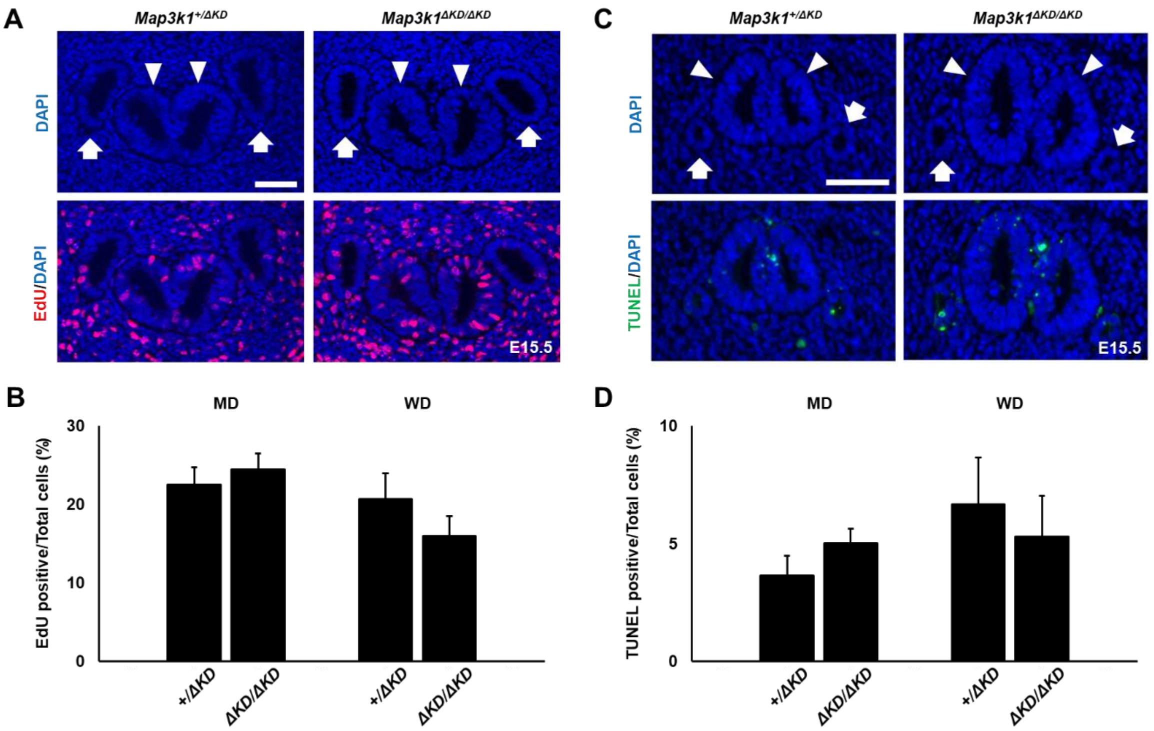

Fig. S2. Cell proliferation and apoptosis in the embryonic reproductive tract. The *Map3k1*$^{+/\Delta KD}$ and *Map3k1* $^{\Delta KD/\Delta KD}$ embryos at E15.5 were subjected to (A and B) detection of EdU labeling for cell proliferation or (C and D) TUNEL staining for apoptosis. (A and C) Images of the stained sections and (B and D) quantifications of positive cells in the Wolffian duct (WD, arrows) and Müllerian duct (MD, arrowheads) epithelium. Values are mean ± s.e.m. of 3 embryos/genotype. Scale bars, 50 µm in A and C.

Disease Models & Mechanisms • Supplementary information

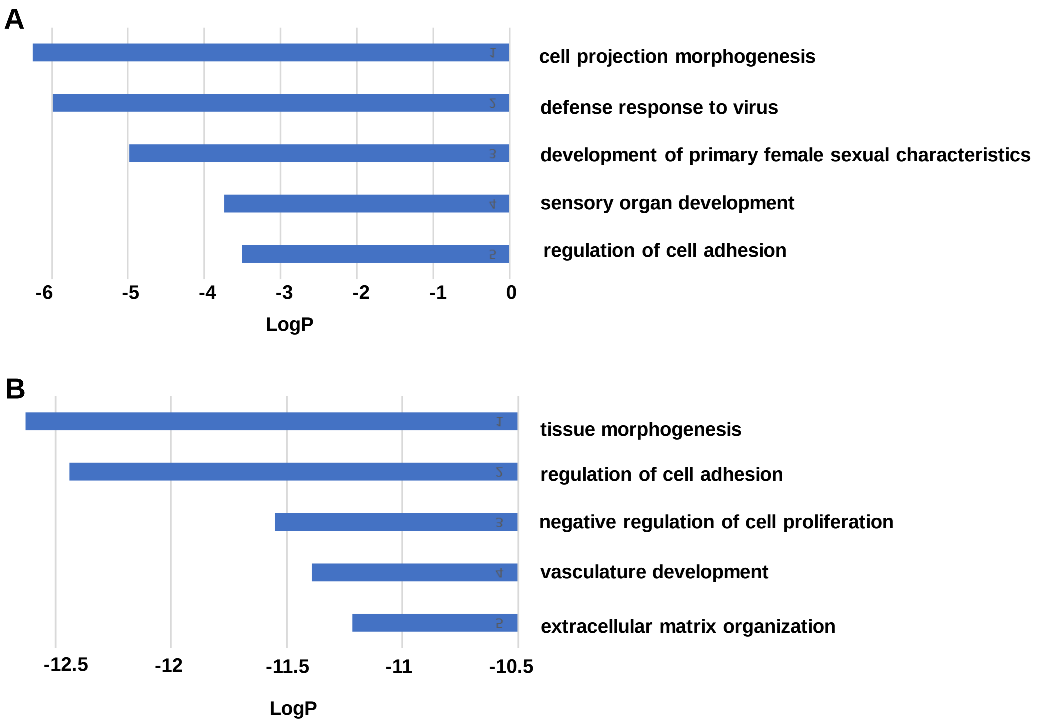

Fig. S3. MAP3K1 promotes tissue morphogenesis. Enrichment analyses of MAP3K1-up-regulated genes in (A) SAM vs shRNA HaCaT cells, and (B) wild type vs *Map3k1*-knockout mouse keratinocytes, identified the top up-regulated biological functions.

Disease Models & Mechanisms • Supplementary information

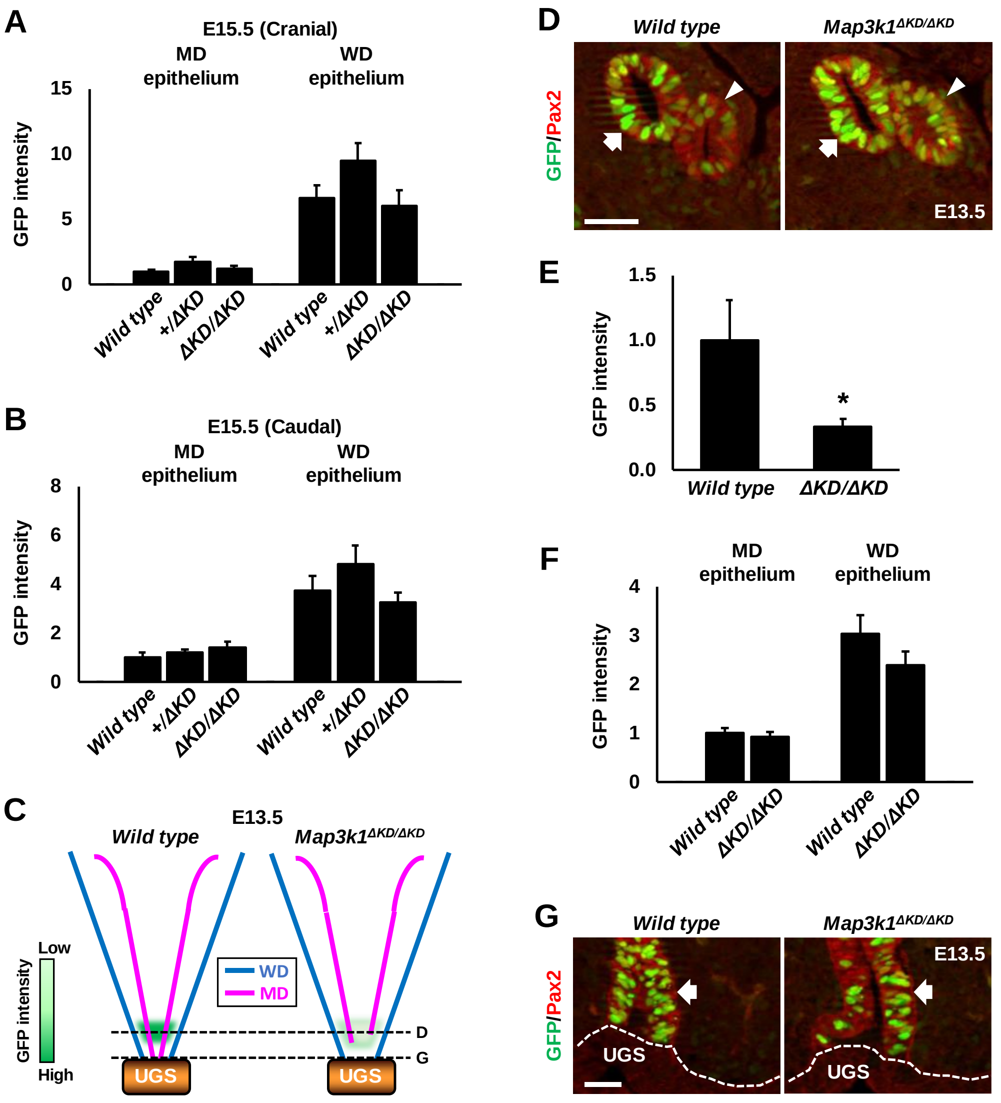

Fig. S4. WNT signaling activity in the embryonic reproductive tract. GFP intensity in the Müllerian duct (MD) and Wolffian duct (WD) epithelium of (A) cranial and (B) caudal sections were quantified in E15.5 TCF/Lef:H2B-GFP wild type, *Map3k1*$^{+/\Delta KD}$ and *Map3k1*$^{\Delta KD/\Delta KD}$ embryos. (C) Diagrammatic illustration of the observed GFP signal intensity in E13.5 TCF/Lef:H2B-GFP wild type and *Map3k1*$^{\Delta KD/\Delta KD}$ embryos. Dotted lines mark the relative positions of the transverse sections shown in D and G. The tissue sections were immunohistochemically stained with anti-Pax2 to mark epithelium of the developing reproductive tract. (D) The images of the caudal reproductive tract. The GFP signal intensity was quantified in (E) mesenchyme and (F) MD and WD epithelium of the caudal reproductive tract. (G) Representative images of the UGS sections. Scale bars, 50 µm. Arrows, WD; arrowheads, MD. Values are mean ± s.e.m., *p<0.05 is significantly different between wild type and *Map3k1*$^{\Delta KD/\Delta KD}$ embryos.

Disease Models & Mechanisms • Supplementary information

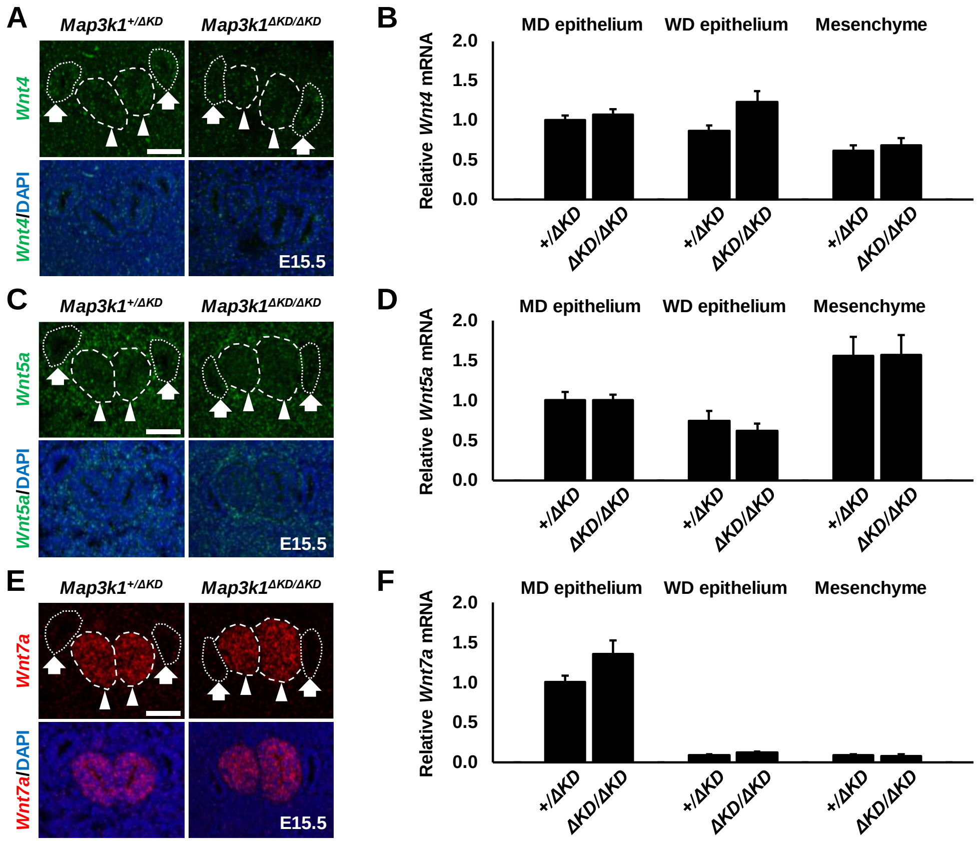

Fig. S5. WNT ligand expression in the embryonic reproductive tract. RNAscope of transverse reproductive tract sections probed for (A and B) *Wnt4*, (C and D) *Wnt5a*, and (E and F) *Wnt7a* mRNAs in *Map3k1*$^{+/\Delta KD}$ and *Map3k1*$^{\Delta KD/\Delta KD}$ E15.5 embryos. (A, C and E) Representative fluorescent images. Scale bars, 50 µm. Arrows, Wolffian ducts (WD); arrowheads, Müllerian ducts (MD). (B, D and F) Quantification of fluorescent signals in MD and WD epithelium and surrounding mesenchyme. Values are mean ± s.e.m.

Disease Models & Mechanisms • Supplementary information

Table S1. Chemicals and Reagents

Name	Company/address	Cat. No
VECTASHIELD	Vector Laboratories, Burlingame, CA	H-1000
Bovine Serum Albumin (BSA)<br>Sucrose<br>Triton X-100<br>2-Mercaptoethanol<br>Boric acid	Sigma-Aldrich, St. Louis, MO	A2153<br>S9378<br>X-100<br>M-7522<br>B-7901
Harris Hematoxylin Stain<br>Eosin-Y Alcoholic Stain	Azer Scientific, Morgantown, PA	ES701<br>ES709
Mounting Medium Xylene	Fisher Health Care, Pittsburgh, PA	245-691
Paraformaldehyde (96%)	Alfa Aesar, UK	A11313.36
N,N,N',N'-Tetrakis(2-hydroxypropyl)ethylenediamine	Thermoscientific, Germany	L16280.AE
Urea<br>Sodium dodecyl sulfate<br>Sodium azide	Fisher Scientific, Fair Lawn, NJ	BP169<br>BP166<br>S227
Zwittergent 3-10 detergent	Millipore, Germany	693021
ApopTag Plus Fluorescein *In Situ* Apoptosis Detection Kit	Millipore, Temecula, CA	S7111
Glycerol	Research Organics, Cleveland, Ohio	5028G
Fetal Bovine Serum (FBS)	R&D System, Flowery Branch, GA	S11150
PureLink RNA Mini Kit	Invitrogen, Carlsbad, CA	12183025
Tissue-Tek O.C.T. Compound	Sakura Finetek Japan, Japan	4583
Glutaldehyde	Sigma-Aldrich, St. Louis, MO	G5882
X-gal	Gold Biotechnology, St. Louis, MO	X4281C10
RNAscope Multiplex Fluorescent Reagent Kit v2-Mm	Advanced Cell Diagnostics, Newark, CA	323280
RNAscope Probe-Mm-Wnt4	Advanced Cell Diagnostics, Newark, CA	401101
RNAscope Probe-Mm-Wnt5	Advanced Cell Diagnostics, Newark, CA	316791
RNAscope Probe-Mm-Wnt7a-C2	Advanced Cell Diagnostics, Newark, CA	401121-C2
RNAscope Probe-Mm-Wnt7b-C2	Advanced Cell Diagnostics, Newark, CA	401131-C2
Dulbecco’s Modification of Eagle’s Medium (DMEM)	Corning, Manassas, VA	10-017-CV
Fetal bovine serum (FBS)	R&D Systems, Flowery Branch, GA	S11150
Puromycin	VWR, Radnor, PA	97064-280
Blasticidin	Thermo Fisher Scientific, Waltham, MA	R21001
Hygromycin	Calbiochem, La Jall, CA	400051

Disease Models & Mechanisms • Supplementary information

Table S2. Primary antibody resources

Target protein	Site of expression	Host	Company	Dilution ratio	Cat. No.
Pax2	MD &WD epithelium	Rabbit	Biolegend	1:100	901001
E-cadherin	Epithelium	Mouse	BD Biosciences	1:100	610182
Acetylated-α tubulin	Apical membrane of MD & WD epithelium	Mouse	Invitrogen	1:100	T7451
Cytokeratin 10	Differentiated epithelium	Rabbit	Biolegend	1:100	905401
Cytokeratin 8	Immature epithelium	Rat	DSHB	1:100	TROMA-I-b
Cytokeratin 14	Basal epithelium	Rabbit	Invitrogen	1:100	PA5-28002
β-catenin	Epithelium	Mouse	BD Biosciences	1:100	610153
Phospho-JNK	Cytosol and/or nucleus	Rabbit	Cell Signaling Technologies	1:100	4668S
Phospho-ERK	Cytosol and/or nucleus	Rabbit	Cell Signaling Technologies	1:100	9101S
Phospho-p38	Cytosol and/or nucleus	Rabbit	Cell Signaling Technologies	1:100	4631S

Table S3. sgRNA sequences

sgRNA	Seq 5'-3'
U6-g5 F	TAAGCAGAAGACATCACCGTGCGGCCGGGACTACC
U6-g5 R	TAAGCAGAAGACATGACGAAAAAAAGCACCGACTCGG
U6-g12 F	TAAGCAGAAGACATCGTCTTTTTTTCGTGGCTGAGCC
U6-g12 R	TAAGCAGAAGACATCCTGCTCCCGGAGAAAGGGTA
U6-g13 F	TAAGCAGAAGACATCAGGGAGGGCCTATTTCCCATGATT
U6-g13 R	TAAGCAGAAGACATCGCCAAAAAAAGCACCGACTC
U6-g14 F	TAAGCAGAAGACATGGCGTTTTTTTCGTGGCTGAGCC
U6-g14 R	TAAGCAGAAGACATCAACCTCCCGGAGAAAGGGTA
U6-g15 F	TAAGCAGAAGACATGTTGGAGGGCCTATTTCCCATGA
U6-g15 R	TAAGCAGAAGACATTAAAACCGTCGTCGGGATTCCC

Table S4. Primer and probe sequences

Gene	Forward sequence (5'-3')	Reverse sequence (5'-3')
TCF4	AGGCTGGAGAAAATACCACAG	AGCAGTTCTCAACCCAAGTG
FZD7	CGAGGCGCTCATGAACAAG	CAGGTAGGGCGCGGTAG
JUN	AAACTTTCCCTGTCAAAGGC	TGAGGGCATCGTCATAGAAG
FOSL1	CCTCAGCTCATCGCAAGAGT	ACATTGGCTAGGGTGGCATC
HOXA10	TGACAAGCACACCACAATTC	TTATTCCGCTTACCCCAGTC
HOXA11	CCAAGTACCAGATCCGAGAG	TGACGATCAGTGAGGTTGAG
HOXA13	TAGGACACTGCGTTAGATGG	ATTTACCTGAGCAGACGCTT
WNT5A	TTTAGAGCGTGTTGCTGTTG	TCAAAGGGCATAGAGACACC
WNT7A	ATCGGGACTATGAACCGGA	CAGGCCTGGGATCTTGTTAC
WNT7B	CCTGGTCCTTTTACCCTGAC	CTCATGCTCCTCAGAGACAG
WNT9B	CGCGAGGAGATGCTAGAG	GAAGGGCGTCAGGACTTC
MAP3K1	AGGCGTCTTTCCCATGATGTCAGT	TCCGCTAAACTGTGGCAAGGAGTA
Hoxa10	CTCCTTTTTGGTCGACTCGC	GCGCTTCATTACGCTTGCT
Hoxa11	GTCTCGGGTCCAGATTTCTC	ACTCTCTGAAGGTCACTTCG
Hoxa13	CCAAATGTACTGCCCCAAAG	CCCGTTCGAGTTCTTTCAAC
Wnt5a	ATGAACTGGGGGCATCTTG	CTCTCTAACGTCCATCAGCG
Wnt7a	CTTGTTGCGCTTGTTCTCC	GGGCAATCCACATAGCCTG
Wnt7b	GCCTAGTGTCCACTGATACC	GGAGGAAAGGGTGGGATATG
Wnt9b	GAGGAGATGCGAGAGTGC	GAAGGGTGTCAGGACCTC
Map3k1	TGGGTCACCTCAACCATCCAAACA	TTCTCGTGGAGATAGGAAAGGCCA

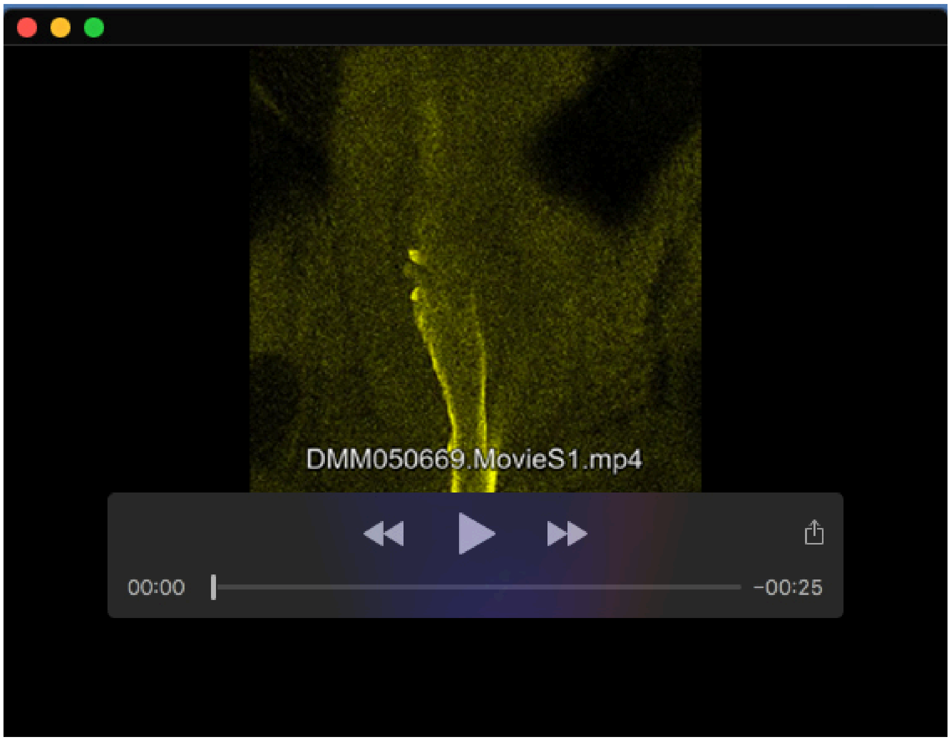

Movie 1. Longitudinal view video of Keratin 14 staining of the vagina in a *Map3k1*$^{+/\Delta KD}$ neonate, corresponding to the *Map3k1*$^{+/KD}$ pups in Fig. 2A (left).

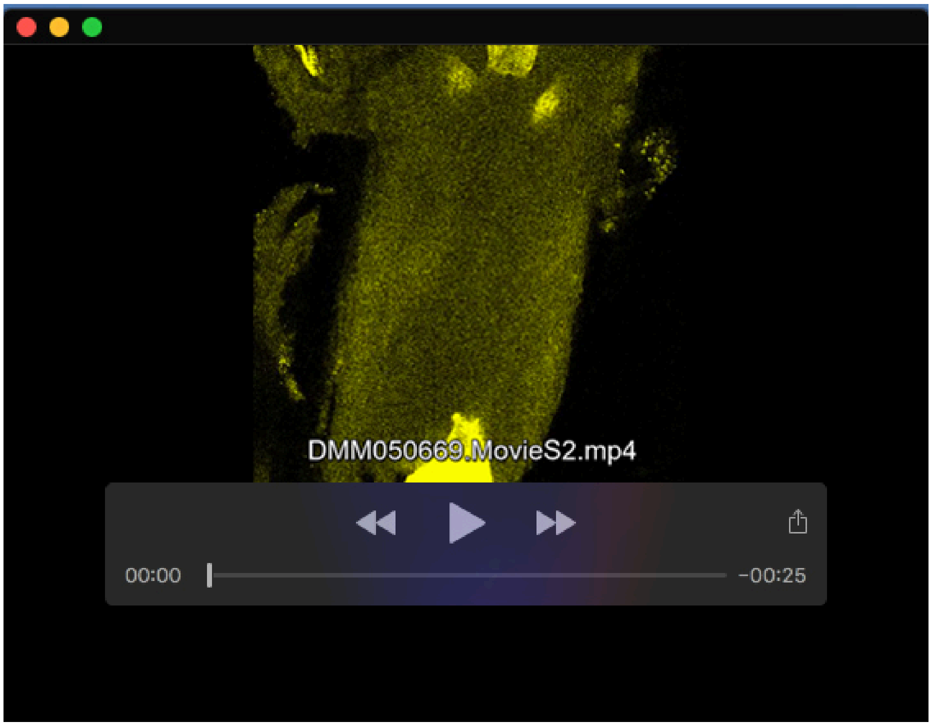

Movie 2. Longitudinal view video showing Keratin 14 staining of the vagina in a *Map3k1*$^{\Delta KD/\Delta KD}$ neonate, corresponding to the distorted female reproductive tract in Fig. 2A (middle).

Disease Models & Mechanisms • Supplementary information

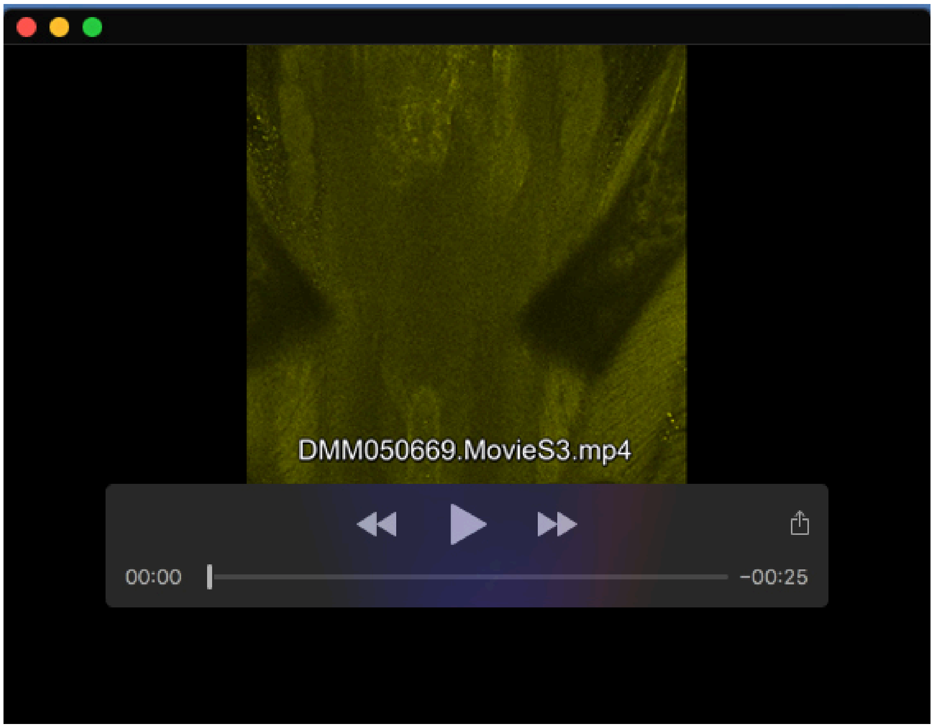

Movie 3. Longitudinal view video showing Keratin 14 staining of the vagina in a *Map3k1*$^{\Delta KD/\Delta KD}$ neonate, corresponding to the stunted female reproductive tract in Fig. 2A (right).

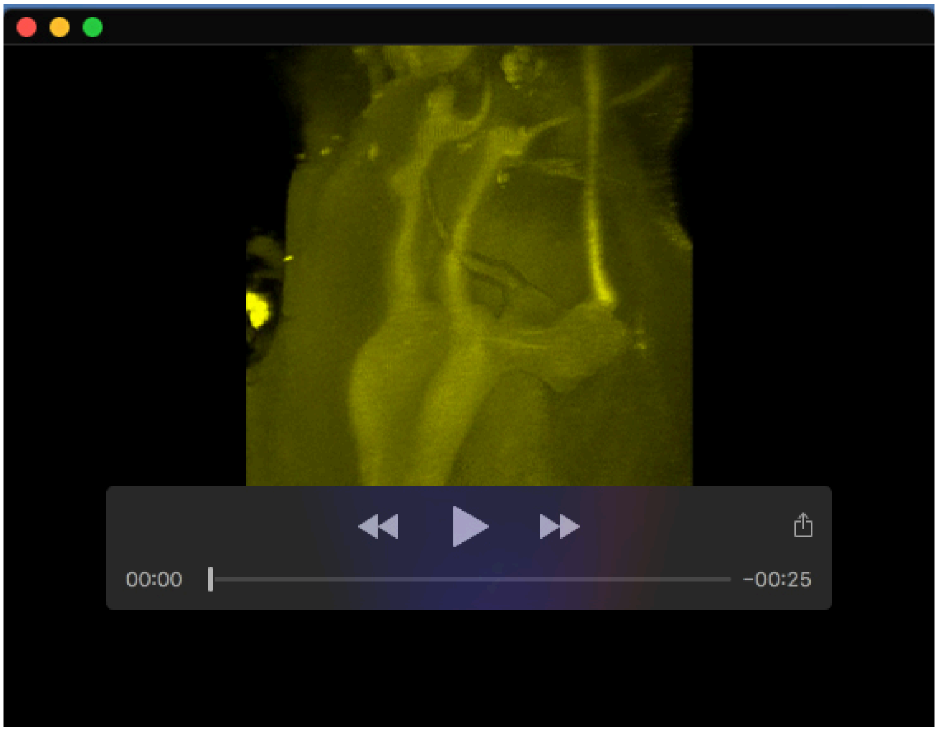

Movie 4. 3D rotation video showing Keratin 8 staining of the developing urogenital system in an E15.5 *Map3k1*$^{+/\Delta KD}$ embryo, corresponding to the female *Map3k1*$^{+/\Delta KD}$ embryo in Fig. 3A.

Disease Models & Mechanisms • Supplementary information

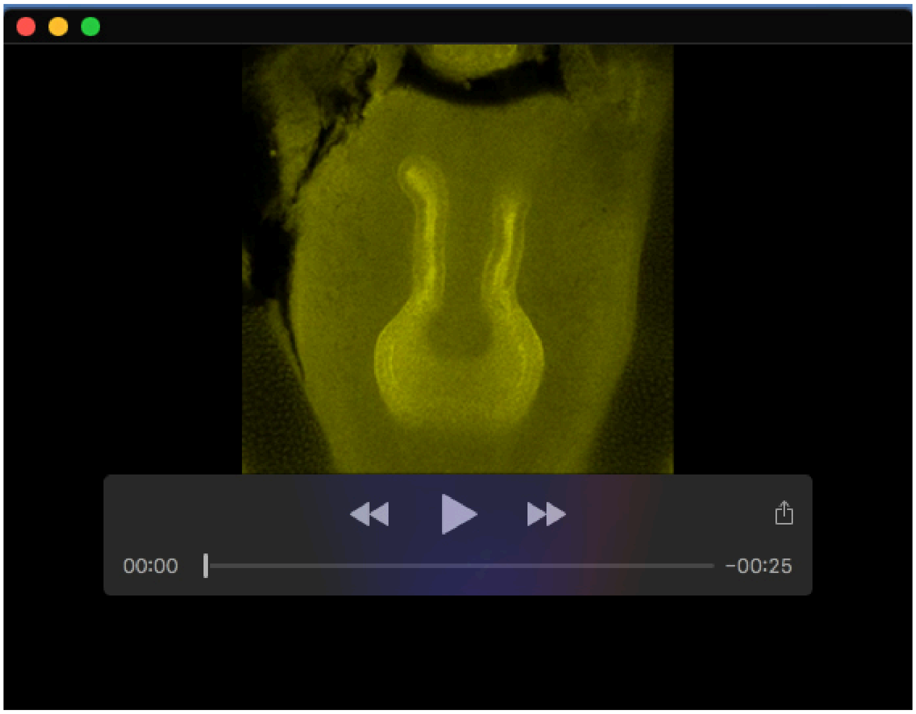

Movie 5. Transverse view video showing Keratin 8 staining of the developing urogenital system in an E15.5 *Map3k1*$^{+/\Delta KD}$ embryo, corresponding to the female *Map3k1*$^{+/\Delta KD}$ embryo in Fig. 3A.

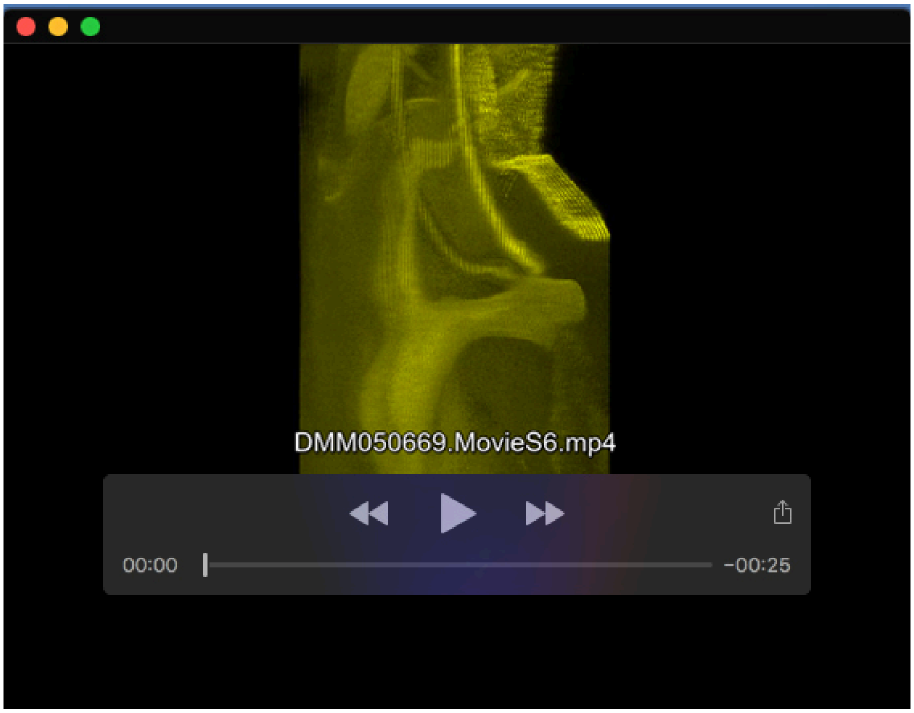

Movie 6. 3D rotation video showing Keratin 8 staining of the developing urogenital system in an E15.5 *Map3k1*$^{\Delta KD/\Delta KD}$ embryo, corresponding to the female *Map3k1*$^{\Delta KD/\Delta KD}$ embryo in Fig. 3A.

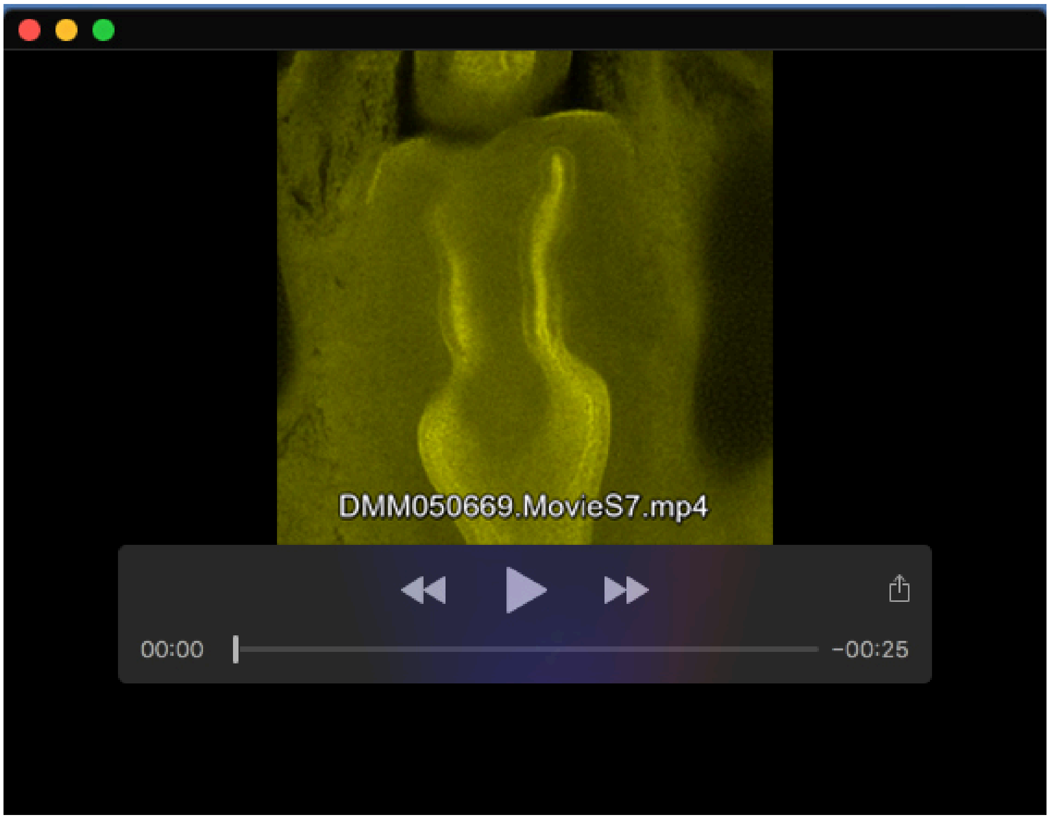

Movie 7. Transverse view video showing Keratin 8 staining of the developing urogenital system in an E15.5 *Map3k1*$^{\Delta KD/\Delta KD}$ embryo, corresponding to the female *Map3k1*$^{\Delta KD/\Delta KD}$ embryo in Fig. 3A.
